# Age-, sex- and ethnicity-related differences in body weight, blood pressure, HbA_1c_ and lipid levels at the diagnosis of type 2 diabetes relative to people without diabetes

**DOI:** 10.1007/s00125-020-05169-6

**Published:** 2020-05-21

**Authors:** Alison K. Wright, Paul Welsh, Jason M. R. Gill, Evangelos Kontopantelis, Richard Emsley, Iain Buchan, Darren M. Ashcroft, Martin K. Rutter, Naveed Sattar

**Affiliations:** 1 Division of Diabetes, Endocrinology and Gastroenterology, School of Medical Sciences, University of Manchester, Manchester, UK; 2 Centre for Pharmacoepidemiology and Drug Safety, Division of Pharmacy and Optometry, School of Health Sciences, Manchester Academic Health Sciences Centre, University of Manchester, Manchester, UK; 3 Institute of Cardiovascular & Medical Sciences, BHF Glasgow Cardiovascular Research Centre, University of Glasgow, 126 University Place, Glasgow, G12 8TA UK; 4 Division of Population Health, Health Services & Primary Care, School of Health Sciences, University of Manchester, Manchester, UK; 5 Department of Biostatistics & Health Informatics, Institute of Psychiatry, Psychology and Neuroscience, King’s College London, London, UK; 6 Department of Public Health and Policy, Institute of Population Health Sciences, University of Liverpool, Liverpool, UK; 7 Manchester Diabetes Centre, Manchester Academic Health Sciences Centre, Manchester University NHS Foundation Trust, Manchester, UK

**Keywords:** Age, Blood pressure, Ethnicity, HbA_1c_, Sex, Weight

## Abstract

**Aims/hypothesis:**

The aim of this work was to determine how weight patterns together with blood glucose, BP and lipids vary at diagnosis of diabetes by age, sex and ethnicity.

**Methods:**

Using the UK Clinical Practice Research Datalink, we identified people with type 2 diabetes (*n* = 187,601) diagnosed in 1998–2015 and compared their weights, HbA_1c_, BP and lipid levels at diagnosis with age-matched people without diabetes (*n* = 906,182), by sex and ethnic group.

**Results:**

Younger age at diagnosis was associated with greater adjusted mean difference (95% CI) in weight between those with vs without type 2 diabetes: 18.7 (18.3, 19.1) kg at age 20–39 years and 5.3 (5.0, 5.5) kg at age ≥ 80 years. Weight differentials were maximal in white women, and were around double in white people compared with South Asian and black people. Despite lower absolute values, BP differences were also greater at younger age of diabetes onset: 7 (6, 7) mmHg at age 20–39 years vs −0.5 (−0.9, −0.2) at age ≥ 80 years. BP differences were greatest in white people, and especially in women. Triacylglycerol level differences were greatest in younger men. Finally, HbA_1c_ levels were also higher with younger onset diabetes, particularly in black people.

**Conclusions/interpretation:**

At diagnosis of type 2 diabetes, when compared with people without diabetes, weight and BP differentials were greater in younger vs older people, in women vs men and in white vs South Asian and black people. These differences were observed even though South Asian and black people tend to develop diabetes a decade earlier with either similar or greater dysglycaemia. These striking patterns may have implications for management and prevention.

**Figure:**
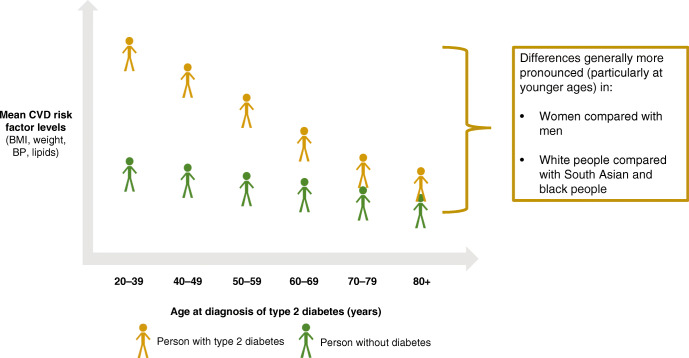
Graphical abstract

**Electronic supplementary material:**

The online version of this article (10.1007/s00125-020-05169-6) contains peer-reviewed but unedited supplementary material, which is available to authorised users.

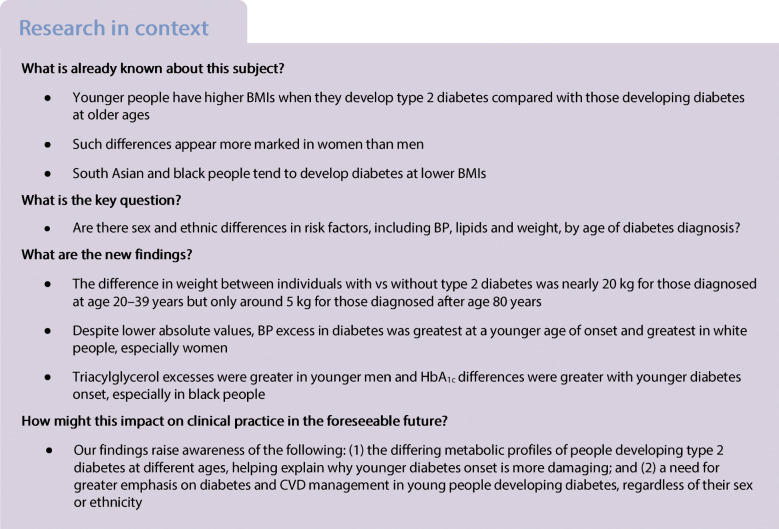



## Introduction

Recent studies show that diagnosis of type 2 diabetes at younger ages is associated with greater excess CVD risk than when the diagnosis occurs later in life [1, 2]. Diagnosis of type 2 diabetes before the age of 40 years is associated with a 10 year reduction in life expectancy, on average, compared with age-matched people without diabetes. However, this excess risk gradually attenuates when diabetes is diagnosed at older ages, such that there is a negligible effect on life expectancy when diagnosis occurs after 80 years of age [3–5]. There are also historical data suggesting that excess risk of cardiovascular events and all-cause mortality from type 2 diabetes is greater in women than in men [6–9]. We also recently observed that for a given age of diagnosis the number of life-years lost due to type 2 diabetes was lower in South Asian and black people compared with white Europeans [5]. This is somewhat surprising and may reflect earlier CVD risk factor management in people from minority ethnic groups as they tend to develop diabetes earlier than white people, or it may reflect other poorly understood factors.

There is a paucity of data on characteristics of individuals at the time of diagnosis of diabetes and how this might have changed over time. Some data indicate that clinical characteristics at the time of developing type 2 diabetes vary by age, sex and ethnicity [10–17]. One such variable is BMI, with studies reporting that it is much higher at diabetes diagnosis in younger vs older people, in women vs men, and in people of white European descent vs other ethnicities [15–17]. There are also some data suggesting more adverse lipid profiles and higher BP levels in people who develop diabetes at younger ages vs non-diabetic age- and sex-matched adults, and in women vs men [10–13]. The majority of these studies were limited in their generalisability due to the data source or specific populations considered and they did not include ethnically diverse populations or only observed risk factor levels in people with diabetes.

We hypothesise that at the time of type 2 diabetes diagnosis, CVD risk factors would be higher when compared with those in people without diabetes and that these differences would vary meaningfully by age, sex and ethnicity. We aimed to determine whether differences in risk factors could better explain contrasting risks for developing type 2 diabetes and its complications. These data are important because they may help focus where more aggressive care is needed to prevent and manage type 2 diabetes.

## Methods

### Data source

We used data from the Clinical Practice Research Datalink (CPRD), an anonymised, longitudinal primary care medical record database from participating UK general practices [18]. Individuals included in the CPRD are broadly representative of the general population in terms of age, sex and ethnicity [18]. The CPRD dataset was linked, at patient-level, to hospitalisation data (Hospital Episode Statistics [HES]), national mortality data (Office for National Statistics [ONS]) and deprivation data (Index of Multiple Deprivation [IMD] 2010) for all eligible patients in 383 linkage-consenting English general practices.

### Study population

We identified a cohort of individuals with incident diabetes (*N* = 193,952), from Read codes (https://digital.nhs.uk/article/1104/Read-Codes) in the electronic health record; the cohort included those for whom the first diagnostic code for diabetes (type 1 or type 2 diabetes) was recorded between 1 January 1998 and 31 March 2015, with no diagnoses prior to this date. The index date was taken as the date of the first diabetes diagnostic Read code.

The cohort of individuals identified with incident diabetes were initially assigned to ‘definite’, ‘probable’ and ‘possible’ type 1 diabetes or type 2 diabetes groups. Due to the nature of primary care data and recording, misclassification, misdiagnosis and miscoding can occur [19]. To try and mitigate these errors, we used a validated algorithm for use in routinely collected data that re-classifies type 1 and type 2 diabetes based on diagnostic codes, glucose-lowering drugs, age, BMI and ethnicity [20]. The implementation of the algorithm (including detailed patient numbers for each step) in this population has been described previously [5]. Only those with a final classification of type 2 diabetes (*N* = 187,968) were considered (type 1 diabetes *N* = 5984). Additionally, people aged less than 20 years at the index date were excluded, resulting in a final type 2 diabetes cohort of 187,601.

Individuals with type 2 diabetes were matched with up to five control individuals without diabetes who were currently registered and contributing data at the cases’ index date, by year of birth (±2 years), sex, general practice and index date. All individuals were observed from the index date to study end (31 March 2015), practice’s last data collection date, death or transfer out of practice, whichever occurred earliest.

### Cohort demographics and baseline characteristics

Age, as defined at the index date, was categorised into six groups: 20–39 years; 40–49 years; 50–59 years; 60–69 years; 70–79 years; and ≥ 80 years. Ethnicity was identified from primary care records and through linkage with HES; the classification of ethnicity using the two data sources has been described previously and further details are provided in electronic supplementary materials (ESM) Methods and ESM Fig. 1 [5]. Ethnicity was categorised into five groups: white, South Asian, black/black British, other and unknown. Deprivation data was defined using the IMD 2010, a national scheme based on seven deprivation domains and available at small-area level to link with the address of the patient, categorised into five quintiles: IMD 1 (least deprived) to IMD 5 (most deprived) [21]. Drug prescriptions (issue of prescription) at baseline were defined as a prescription within 90 days before or after the index date. Cardiovascular disease (myocardial infarction, stroke, CHD, peripheral vascular disease, angina pectoris) and renal disease (chronic kidney disease stage 4 and above) were defined by Read code, up to the index date.

### Outcome measures

Body weight, BMI, systolic BP and lipid levels (total cholesterol, HDL-cholesterol, non-HDL-cholesterol, triacylglycerol) were defined as the closest measure up to 12 months before and after the index date (this time window was used to improve capture of available data, particularly for people without diabetes). HbA_1c_ was only examined in people with type 2 diabetes due to the proportion of missing data in those without diabetes.

### Statistical analysis

Descriptive characteristics for people with type 2 diabetes and matched individuals without diabetes were summarised using mean (±SD) and proportions as appropriate.

A multivariate imputation by chained equations (MICE) algorithm was used to impute missing data on baseline variables (BMI, weight, BP, cholesterol, triacylglycerol). Details on the proportion of missingness and differences in characteristics between those with and without missing data are provided in the ESM Tables 1–4 and ESM Key differences in missingness. Imputation models were estimated separately for people with type 2 diabetes and control-group individuals without diabetes within age, age–sex and age–ethnicity strata. See ESM Imputation model for a list of variables included in the imputation model. Five imputed datasets were generated. Analyses comparing biological outcome measures between the type 2 diabetes and control groups were stratified by age, age–sex and age–ethnicity. Multiple linear regression, adjusting for deprivation and accounting for matching, was used to calculate the adjusted mean differences (95% CI) in biological variables between those with and without type 2 diabetes across age groups. Estimates were combined across the five datasets using Rubin’s rules [22]. *p*< 0.05 was considered statistically significant. All statistical analyses were performed using Stata 15.1 (StataCorp, USA).

## Results

**Fig. 1:**
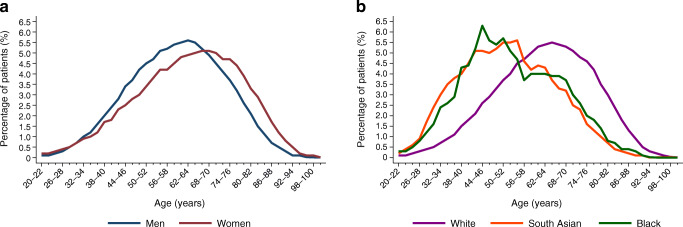
Age distribution at diagnosis of type 2 diabetes by sex (**a**) and ethnicity (**b**)

Baseline characteristics of people with type 2 diabetes are presented in Table 1 and those of control groups without diabetes are presented in ESM Table 5. The cohort comprised 187,601 people with incident type 2 diabetes (mean ± SD age 61.9 ± 14.1 years; 55.2% male sex; 76.5% white) and 906,182 individuals without diabetes as matched controls. People with type 2 diabetes were more likely than control individuals to be obese, to have high BP and to be prescribed antihypertensive, lipid-lowering and antiplatelet agents. At diagnosis of type 2 diabetes, women were on average 3 years older than men (Fig. 1a) and had higher mean BMI and cholesterol and lower HbA_1c_. Women were less likely than men to receive lipid-lowering and antiplatelet medication but were more likely to receive antihypertensive agents (Table 1).

**Table 1:** Baseline characteristics of people with incident type 2 diabetes

Characteristic	All	Sex		Ethnicity		
		Male	Female	White	South Asian	Black
*N*	187,601	103,607	83,994	143,481	9486	4451
Men, *n* (%)	103,607 (55.2)			78,573 (54.8)	5142 (54.2)	2204 (49.5)
Age, years	61.9 ± 14.1	60.4 ± 13.4	63.7 ± 14.8	63.3 ± 13.8	52.6 ± 13.5	54.0 ± 13.8
Age group, *n* (%)						
20–39 years	11,997 (6.4)	6779 (6.5)	5218 (6.2)	7170 (5.0)	1737 (18.3)	650 (14.6)
40–49 years	25,770 (13.7)	15,949 (15.4)	9821 (11.7)	16,956 (11.8)	2310 (24.4)	1179 (26.5)
50–59 years	41,491 (22.1)	25,186 (24.3)	16,305 (19.4)	30,162 (21.0)	2464 (26.0)	1075 (24.2)
60–69 years	48,469 (25.8)	27,917 (27.0)	20,552 (24.5)	38,539 (26.9)	1837 (19.4)	868 (19.5)
70–79 years	39,452 (21.0)	19,876 (19.2)	19,576 (23.3)	33,198 (23.1)	934 (9.9)	528 (11.9)
≥ 80 years	20,422 (10.9)	7900 (7.6)	12,552 (14.9)	17,456 (12.2)	204 (2.2)	151 (3.4)
Ethnicity, *n* (%)						
White	143,481 (76.5)	78,573 (75.8)	64,908 (77.3)			
South Asian	9486 (5.1)	5142 (5.0)	4344 (5.2)			
Black	4451 (2.4)	2204 (2.1)	2247 (2.7)			
Other	2480 (1.3)	1366 (1.3)	1114 (1.3)			
Unknown	27,703 (14.8)	16,322 (15.8)	11,381 (13.6)			
Deprivation (IMD 2010), *n* (%)						
IMD 1 (least)	34,290 (18.3)	19,750 (19.1)	14,540 (17.3)	26,192 (18.3)	1238 (13.1)	222 (5.0)
IMD 2	40,934 (21.8)	23,177 (22.4)	17,757 (21.1)	32,691 (22.8)	1405 (14.8)	365 (8.2)
IMD 3	38,5337 (20.5)	21,443 (20.7)	17,094 (20.4)	29,504 (20.6)	1963 (20.7)	713 (16.0)
IMD 4	39,128 (20.9)	21,121 (20.4)	18,007 (21.4)	29,432 (20.5)	2300 (24.3)	1440 (32.4)
IMD 5 (most)	34,379 (18.3)	17,956 (17.3)	16,423 (19.6)	25,420 (17.7)	2566 (27.1)	1703 (38.3)
Unknown	333 (0.2)	160 (0.2)	173 (0.2)	242 (0.2)	14 (0.2)	8 (0.2)
CVD, *n* (%)^a^	37,765 (20.1)	23,453 (22.6)	14,312 (17.0)	32,255 (22.5)	1191 (12.6)	359 (8.1)
Renal disease (CKD stage ≥4), *n* (%)	2335 (1.2)	1257 (1.2)	1078 (1.3)	1943 (1.4)	109 (1.2)	55 (1.2)
Biological variables (±12 months from diagnosis)						
BMI, kg/m^2^	31.3 ± 6.9	31.1 ± 6.2	31.6 ± 7.6	31.6 ± 6.9	28.5 ± 5.8	31.1 ± 6.6
Missing, %	18.6	17.7	19.6	17.9	23.3	24.0
Weight, kg	88.9 ± 21.1	94.0 ± 19.9	82.4 ± 20.6	89.9 ± 21.2	77.4 ± 16.3	87.7 ± 19.0
Missing, %	17.0	16.2	17.9	16.4	21.4	22.4
Total cholesterol, mmol/l	5.2 ± 1.3	5.0 ± 1.3	5.3 ± 1.3	5.2 ± 1.3	5.1 ± 1.2	5.1 ± 1.2
Missing, %	16.8	15.9	18.0	16.2	20.6	22.4
HDL-cholesterol, mmol/l	1.2 ± 0.4	1.1 ± 0.3	1.3 ± 0.4	1.2 ± 0.4	1.2 ± 0.3	1.3 ± 0.4
Missing, %	28.9	28.1	29.9	28.6	28.8	30.6
Non-HDL-cholesterol, mmol/l	4.0 ± 1.3	3.9 ± 1.3	4.0 ± 1.3	3.9 ± 1.3	3.9 ± 1.2	3.8 ± 1.2
Missing, %	29.0	28.2	29.9	28.6	28.9	30.6
Triacylglycerol, mmol/l	2.6 ± 2.6	2.5 ± 3.0	2.1 ± 1.9	2.3 ± 2.5	2.3 ± 3.4	1.5 ± 1.5
Missing, %	29.9	28.8	31.3	29.9	28.4	29.3
BP						
Systolic, mmHg	140 ± 19	139 ± 18	140 ± 20	140 ± 19	133 ± 18	138 ± 19
Diastolic, mmHg	81 ± 11	81 ± 11	80 ± 11	81 ± 11	81 ± 11	82 ± 11
Missing, %	12.5	12.4	12.7	11.9	18.1	18.3
HbA_1c_						
mmol/mol	62 ± 22	63 ± 23	61 ± 21	61 ± 22	65 ± 22	68 ± 27
%	7.8 ± 2.0	7.9 ± 2.1	7.7 ± 2.0	7.8 ± 2.0	8.1 ± 2.0	8.4 ± 2.4
Missing (%)	16.7	16.5	17.0	16.1	21.4	22.9
Drug prescriptions, %						
Diabetes medication						
Any oral hypoglycaemic	44.1	44.7	43.4	43.3	47.6	51.6
Metformin	34.6	35.1	33.9	33.7	39.2	40.9
Sulfonylurea	11.0	11.3	10.7	10.7	11.9	13.4
Insulin	3.6	3.6	3.6	3.6	3.2	6.2
Other	1.6	1.6	1.7	1.6	2.2	2.0
Antihypertensive agent						
Any	54.9	53.0	57.2	57.8	36.5	42.8
α-Blocker	4.3	4.5	4.0	4.6	2.3	5.1
Angiotensin-2 receptor blocker	8.7	7.7	9.9	9.1	8.0	7.5
ACE inhibitor	29.4	31.2	27.2	30.9	19.5	20.5
β-Blocker	19.4	19.5	19.2	21.0	11.1	10.2
Calcium-channel blocker	20.6	20.5	20.7	21.2	14.4	26.5
Diuretic (thiazide, potassium-sparing or loop)	27.5	22.2	34.0	29.8	11.8	19.6
Lipid-lowering therapy (any)	44.1	46.0	41.8	46.0	37.1	30.6
Antiplatelets (any)	26.0	27.6	23.9	28.1	18.5	15.2

Data are mean ± SD, *n* (%) or %, where indicated

^a^CVD includes myocardial infarction, stroke, CHD, peripheral vascular disease and angina pectoris

CKD, chronic kidney disease

Compared with white people, South Asian and black people were on average ~ 9–10 years younger at the onset of type 2 diabetes and had a higher level of deprivation (Table 1, Fig. 1b). Black people and particularly South Asian people developed type 2 diabetes at a substantially lower mean weight/BMI than white people and had lower systolic BP levels but higher HbA_1c_, on average. Correspondingly, compared with white people, a higher percentage of South Asian and black people received prescriptions for glucose-lowering medications and lower proportions were prescribed lipid-lowering, antihypertensive and antiplatelet medications.

Biological variables at age of type 2 diabetes onset were compared against those for control individuals at the same age without diabetes (ESM Table 6). Younger age at type 2 diabetes diagnosis was associated with higher mean body weight in individuals with diabetes and a greater differential compared with individuals without diabetes. As an illustration, in people aged 20–39 years, the mean ± SD weight was 99.7 ± 27.3 kg for those with type 2 diabetes and 81.0 ± 22.5 kg for those without diabetes (adjusted mean [95% CI] difference 18.7 [18.3, 19.1] kg). However, in people aged ≥80 years, the mean ± SD weight was 72.1 ± 17.0 kg in those with type 2 diabetes and 66.9 ± 18.5 kg for those without diabetes (adjusted mean [95% CI] difference 5.3 [5.0, 5.5] kg). A similar age-related pattern was observed for BMI, systolic BP and triacylglycerol. With the exception of systolic BP levels, higher mean levels of risk factors were observed for people in whom type 2 diabetes was diagnosed at a younger vs older age. Larger differences between those with and without type 2 diabetes were consistently observed in younger age groups, with a narrowing in risk factor levels in older age groups. Whilst this was the case for systolic blood pressure, with the largest difference at ages 20–39 (adjusted mean difference 6 [6, 7] mmHg), in those aged ≥80 years, blood pressure values in those with type 2 diabetes exceeded those in individuals without diabetes (adjusted mean difference −0.5 [−0.9, −0.2] mmHg).

**Fig. 2:**
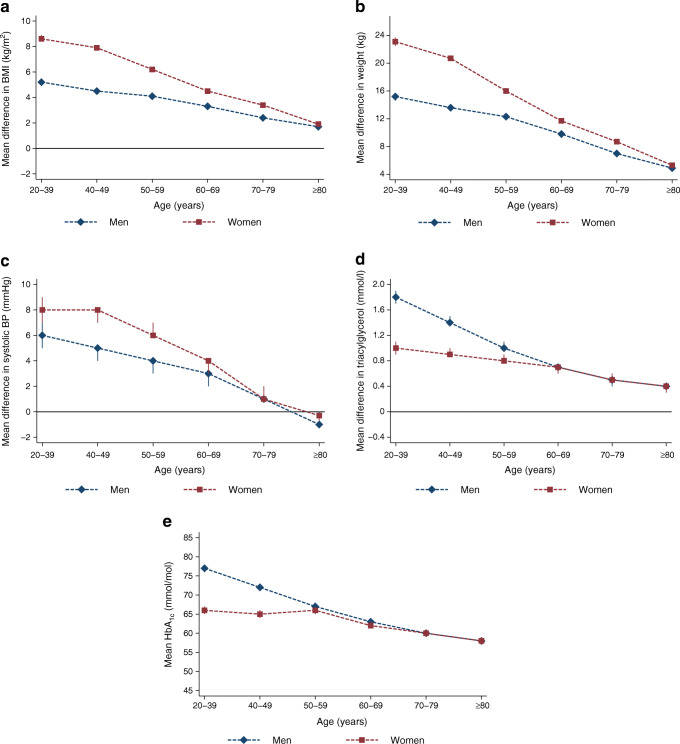
(**a**–**d**) Adjusted age-specific mean (95% CI) differences in BMI (**a**), weight (**b**), systolic BP (**c**) and triacylglycerol level (**d**) in men and women recently diagnosed with type 2 diabetes compared with men and women without diabetes. (**e**) Age-specific mean HbA_1c_ levels in men and women recently diagnosed with type 2 diabetes

This relationship between age and biological variables was observed across sexes. BMI, weight, systolic BP and triacylglycerol differences between those with and without type 2 diabetes were substantially greater in women than in men, particularly at younger ages (Fig. 2a–d and ESM Table 7). For weight, the adjusted mean difference (95% CI) in women aged 20–39 years was 23.1 (22.5, 23.7) kg, reducing to 5.3 (5.0, 5.6) kg in women aged ≥80 years. In men, the adjusted mean difference at age 20–39 years was 15.2 (14.6, 15.7) kg and 4.9 (4.5, 5.3) kg at age ≥ 80 years.

**Fig. 3:**
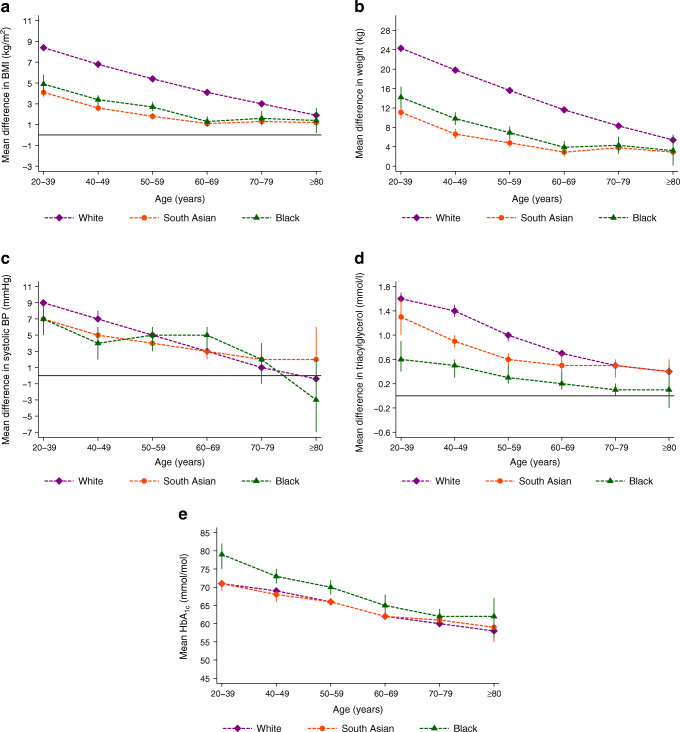
Adjusted age-specific mean (95% CI) differences in BMI (**a**), weight (**b**), systolic BP (**c**) and triacylglycerol level (**d**) in white, South Asian and black people recently diagnosed with type 2 diabetes compared with people without diabetes. (**e**) Age-specific mean HbA_1c_ levels in white, South Asian and black people recently diagnosed with type 2 diabetes ≥ m^2^

Across age groups, there were marked ethnic differences in weight, BMI, BP and lipid levels (ESM Table 8). White people with type 2 diabetes were more likely to have higher levels for these risk factors at diagnosis than South Asian and black people. The differences between those with and without type 2 diabetes was greatest in white people, except for systolic blood pressure where this was lost in those aged ≥50 years (Fig. 3a–d and ESM Table 8). With the exception of people aged ≥80 years, across all age bands, the mean weight difference (95% CI) between people age 20–39 years with type 2 diabetes and control individuals of white ethnicity was approximately double that observed in South Asian and black people: 24.3 (23.8, 24.9) kg in white people; 11.1 (9.8, 12.3) kg in South Asian people; and 14.2 (11.9, 16.4) kg in black people. Similarly, for BMI at age 20–39 years, the mean difference (95% CI) was 8.4 (8.2, 8.5) kg/m^2^ in white people, 4.1 (3.7, 4.5) kg/m^2^ in South Asian people and 4.9 (4.1, 5.8) kg/m^2^ in black people. For systolic BP and triacylglycerol, the differential between those with and without diabetes was greater in white people than in the other two ethnic groups only up to the ages of 49 years and 69 years, respectively.

While we only examined HbA_1c_ in those with new-onset diabetes, these data showed markedly higher mean HbA_1c_ at younger ages, more so in men than in women and in black people compared with the two other ethnicities across nearly all age groups (Figs. 2e, 3e).

The same patterns were observed across ethnic–sex subgroups (ESM Table 9). The impact of diabetes on BMI, weight and systolic BP was most substantial in white women at any given age compared with South Asian and black men and women and was particularly prominent in younger white women. At age 20–39 years, the mean (95% CI) difference in weight between individuals with and without type 2 diabetes was 27.6 (26.9, 28.3) kg in white women, 13.1 (11.4, 14.7) kg in South Asian women, 16.6 (14.2, 19.0) kg in black women, 20.2 (19.5, 20.9) kg in white men, 7.6 (5.7, 9.4) kg in South Asian men and 11.1 (7.6, 14.7) kg in black men. Similarly, at age 20–39 years, the mean (95% CI) difference in systolic BP between those with and without type 2 diabetes was 9.7 (9.0, 10.3) mmHg in white women, 7.6 (5.7, 9.5) mmHg in South Asian women, 6.8 (4.0, 9.6) mmHg in black women, 7.6 (6.9, 8.3) mmHg in white men, 5.2 (3.7, 6.7) mmHg in South Asian men and 6.1 (3.6, 8.6) mmHg in black men.

## Discussion

In this large contemporary primary care-based study from England, our findings were as follows: (1) there was an association between younger age at type 2 diabetes diagnosis and a higher number of elevated cardiovascular risk factors; (2) the differences in weight, BMI and systolic BP in individuals with vs without type 2 diabetes were more marked in women than in men, especially at younger ages, whereas HbA_1c_ and triacylglycerol tended to be higher in men; (3) white people with type 2 diabetes were more likely to have higher weight, BMI, BP and triacylglycerol levels across all age groups (except for the very oldest groups) than South Asian and black people with type 2 diabetes. This poorer cardiovascular risk factor profile may help explain previous observations that young people diagnosed with type 2 diabetes, across all ethnicities, lose more life-years from diabetes than those diagnosed when older [5]. It also explains why, in historical studies, women had greater increase in their relative risk for CVD than men when they develop diabetes, with high BP being a stronger risk factor for CVD than raised triacylglycerol [5, 23]. These data also might explain our prior finding which linked greater loss of life-years from type 2 diabetes in white vs South Asian and black people [5].

Regarding sex differences, it has been established that women generally have a higher BMI than men when diagnosed with type 2 diabetes [14, 24, 25]. In both men and women, the BMI differences are most marked at younger ages of diagnosis and the differences narrow with diagnosis at older ages. A younger age of onset of obesity leads to a greater cumulative exposure to obesity, which may contribute to a younger age of onset of diabetes [15, 26]. Older people being diagnosed with diabetes at a lower body weight could in part be explained by them having a lower cumulative lifetime exposure to obesity.

Young women appear to undergo larger excess weight gain than men prior to being diagnosed with type 2 diabetes [24, 25]. It has been suggested that women may experience greater adverse metabolic changes than men as they develop diabetes, with a poorer cardiovascular risk factor profile when compared with their non-diabetic counterparts. However, while this has been well documented for BMI, weight differences have not been reported and less is known about the impact on other risk factors, especially across the age spectrum. In our comprehensive comparison of seven cardiovascular risk markers, we identified bigger differences (compared with their non-diabetic counterparts) in weight, systolic BP and HDL-cholesterol in women than in men at the diagnosis of type 2 diabetes at any age but particularly in younger people. The converse was seen with triacylglycerol, with men showing a trend for higher levels at the onset of type 2 diabetes, most notably in the youngest age groups. Understanding these patterns, particularly the sex differences, are important considerations for future cardiovascular risk evaluation. Finally, sex biases in prescribing of medications to manage cardiovascular risk factors in people with diabetes have been identified: women are less likely to be prescribed medications for adverse risk factors and/or established CVD [27–30]. Therefore, greater awareness of sex- and age-related differences in biological factors should prompt appropriate intervention.

Regarding ethnic differences, the South Asian and black people with type 2 diabetes were on average 9–10 years younger than the white population. This may arise from the higher diabetes incidence rates seen in younger age groups or may reflect the age distribution of the different ethnic groups. South Asian and black people were observed to present with a different cardiometabolic risk profile, with typically higher HbA_1c_, lower BMI, and established micro- and macrovascular complications [14, 31–33]. As identified previously by Paul et al. [14], and shown in this cohort, white people with type 2 diabetes had a pattern of presenting with higher BMI at any age compared with South Asian and black people, with larger ethnic differences in BMI levels reported at younger ages. We extend these findings by observing a similar pattern across other risk factors including weight, systolic BP and triacylglycerol in white people, as well as a tendency towards higher HbA_1c_ levels in black people. Furthermore, greater risk factor burden at diagnosis was seen in white people, suggesting that they may undergo a greater deterioration in cardiometabolic risk factors on their pathway to developing diabetes. In particular, white women with type 2 diabetes had the most substantial burden of CVD risk factors when compared with their non-diabetic white counterparts. These findings may, in part, help to explain mechanisms behind findings from our previous research on this cohort wherein white people and particularly white women with type 2 diabetes had a greater likelihood for poorer life expectancy and a greater risk of CVD than South Asian and black women [5]. Earlier treatment of abnormal cardiometabolic risk factors in South Asian and black people may also play a role, given their development of disease at a younger age. As South Asian people are known to have an elevated risk of CVD compared with white Europeans [34], there has been a greater emphasis in these populations, particularly for those with type 2 diabetes, to reduce modifiable risk factors through lifestyle changes and drug management for primary and secondary CVD prevention [35].

This study has several important strengths: (1) we obtained a large cohort of people with type 2 diabetes identified from primary care, reducing the likelihood of significant selection bias; (2) we applied a validated algorithm to identify type 2 diabetes to mitigate any potential miscoding or misclassification from electronic health records; (3) we had clinical and prescribing information on people with and without type 2 diabetes; and (4) we combined data from general practice and hospital records to increase completeness and accuracy of ethnicity information.

Our study has several potential limitations. First, there is the potential for misclassification of diabetes when using routinely collected primary care data without validation from consultation free text. The accuracy of primary care Read codes depends on the team entering them, the clinicians’ time and information-technology skills, certainty of diagnosis and organisational issues [36]. The algorithm proposed and validated by de Lusignan et al. [20] uses additional information beyond Read codes alone. These clinical features have been identified by The Royal College of General Practitioners and others, as being important for differentiating between type 1 and type 2 diabetes [19, 37]; therefore, after applying the algorithm to our cohort, we would expect the prevalence of misclassification of the final diabetes type to be lower than the initial classification. However, any residual misclassification might bias our results towards the null, so our results are likely to be conservative estimates of the true difference between control individuals and people diagnosed with type 2 diabetes. Second, depending on the characteristics of an individual, the assessment of type 2 diabetes prompted by a clinician may represent either screening or diagnostic testing [38, 39]. These are two distinct pathways to determining diabetes status and may result in observations of individuals at different stages of the condition at the time of diagnosis. Third, prescription data in the CPRD only indicate when a prescription was issued but not whether the medication was dispensed or taken as recommended. Fourth, there was a large proportion of missing data for cardiovascular risk factors in people without diabetes. We addressed this through multiple imputation with the imputation model including an extensive range of patient characteristics and auxiliary variables; importantly, we noted no clinically meaningful differences in measurements of biological variables when comparing those with and without any missing data (ESM Tables 2–4). We acknowledge that missing data may have occurred preferentially in people with normal or close to normal risk factors (i.e. missing not at random), possibly leading to bias in the magnitude of the association and an underestimation of the differentials in risk factor levels. Fifth, people without type 2 diabetes and attending primary care for cardiovascular risk factor assessment are likely to have abnormal cardiovascular risk factors or be at risk of diabetes or CVD. Therefore, measured differences between those with and without diabetes are potentially underestimated when compared with a healthier non-diabetic control group. Sixth, due to the availability of clinical data, we allowed for the baseline risk factors to be defined from data captured up to 12 months after the index date. This reduced the proportion of missing data in people without diabetes who may not have measures frequently recorded. The majority of people diagnosed with diabetes will have had information recorded either on or close to this date. However, a small proportion of individuals with diabetes would have risk factors recorded only after clinical intervention. Such intervention would be expected to improve but not normalise abnormal risk factors. Therefore, the observed risk factor differences compared with control counterparts may be somewhat underestimated by our analysis. Seventh, linkage with hospital data is only available for people attending English general practices; therefore, generalisation of our findings to other healthcare systems, other countries and other ethnic groups at high risk of diabetes may be limited. However, we would anticipate broadly similar findings. Finally, we recognise that less frequent screening means diabetes is being diagnosed later in the course of disease in younger people. Notably, this cannot explain BMI differentials by sex (men had higher HbA_1c_ levels but lower weight differences than women) or by ethnicity (HbA_1c_ levels were higher or the same but weight differences were far smaller). It is also unlikely to explain the substantial weight differentials seen across the life course.

Targeted screening and active modification of weight in men and South Asian and black people, who may be at risk of developing type 2 diabetes at lower BMI values, may be warranted to prevent the development of type 2 diabetes. Active intervention to reduce cardiovascular risk factors other than weight is justified in all people with type 2 diabetes and those at high risk for diabetes but these data argue for particular focus in younger people, where perhaps more aggressive risk factor management may be needed despite lower mean blood pressures and lower ten year CVD risks, but high lifetime risks. Further clinical trials are required to assess the degree of weight reduction required to achieve diabetes remission by age and ethnicity.

In conclusion, we have provided perhaps the most comprehensive assessment of age-, sex- and ethnicity-based differences in established CVD risk factors at the diagnosis of type 2 diabetes in a high-income country. The findings may help explain why younger people are more likely to lose additional life-years when they develop diabetes and also perhaps the contrast between outcomes in white people and other ethnicities. Clinically, given that men and people from minority ethnic groups tend to develop diabetes at far lower BMI values, better targeting of these populations to prevent overt type 2 diabetes is warranted. However, in all ethnic groups, risk factor differences, as compared with people without diabetes, are worse at younger ages suggesting a much greater need to improve diabetes care in younger people, regardless of their sex or ethnicity. The high prevalence of abnormal CVD risk factors in younger people developing diabetes suggests that there may be value in performing trials in these individuals evaluating the impact of more aggressive lifestyle and/or pharmacotherapy to promote weight loss and CVD risk reduction.

## Electronic supplementary material

ESM (PDF 870 kb)

## Data Availability

Read codes are publicly available at The ClinicalCodes repository [40] and can be accessed at https://clinicalcodes.rss.mhs.man.ac.uk/. Electronic health records are, by definition, considered ‘sensitive’ data in the UK by the Data Protection Act and cannot be shared via public deposition because of information governance restriction in place to protect patient confidentiality. Access to data is available only once approval has been obtained through the individual constituent entities controlling access to the data. The primary care data can be requested via application to the Clinical Practice Research Datalink (https://www.cprd.com); secondary care data can be requested via application to the Hospital Episode Statistics from the UK Health and Social Care Information Centre (www.hscic.gov.uk/hesdata); and mortality data are available by application to the UK Office for National Statistics (www.ons.gov.uk/ons/index.html).

## Abbreviations

CPRD: Clinical Practice Research Datalink
HES: Hospital Episode Statistics
IMD: Index of Multiple Deprivation
ONS: Office for National Statistics
