# Supplementary material for: Age-, sex- and ethnicity-related differences in body weight, blood pressure, HbA1c and lipid levels at the diagnosis of type 2 diabetes relative to people without diabetes
Source: Diabetologia. 2020 May 21;63(8):1542–53. doi: 10.1007/s00125-020-05169-6 (PMC7351865; doi:10.1007/s00125-020-05169-6)

Electronic Supplementary Material (ESM)

Content

	Page
**ESM Methods**	2
Identifying and classifying ethnicity from primary care and hospital data	2
**Missing data**	3
Key differences in missingness	3
Imputation model	3
**ESM Table 1.** Proportion of people with type 2 diabetes and controls with missing data on risk factors by sex and ethnicity	4
**ESM Table 2.** Differences in clinical characteristics between those with and without missing data on biological risk factors	5-6
**ESM Table 3.** Differences in clinical characteristics between those with and without missing data on biological risk factors, by sex	7-8
**ESM Table 4.** Differences in clinical characteristics between those with and without missing data on biological risk factors, by ethnicity	9-12
**ESM Table 5.** Baseline characteristics of controls without diabetes	13-14
**ESM Table 6.** Adjusted age-specific mean (95% CI) differences in biological risk factor levels in people with newly diagnosed type 2 diabetes compared to matched people without diabetes	15
**ESM Table 7.** Adjusted age-specific mean (95% CI) differences in biological risk factor levels in men and women newly diagnosed with type 2 diabetes compared with people without diabetes	16-17
**ESM Table 8.** Adjusted age-specific mean (95% CI) differences in biological risk factor levels in Whites, South Asians and Blacks newly diagnosed with type 2 diabetes compared with people without diabetes	18-19
**ESM Table 9.** Adjusted mean (95%CI) differences in biological risk factor levels at age of diabetes onset between those newly diagnosed with type 2 diabetes compared with people without diabetes, by ethnicity and sex	20-21
**ESM Fig 1.** Combining ethnicity records from CPRD and hospital records (HES)	22

ESM Methods.

Identifying and classifying ethnicity from primary care and hospital data

Ethnicity records were identified from two sources to increase completeness of available ethnicity information and improve accuracy of the categorisation; (i.) primary care data (CPRD) using Read codes recorded by the general practice and (ii.) hospital data (linked to patients identified in CPRD), recorded by a healthcare professional.

In both general practice and in hospital, patients can have their ethnicity recorded repeatedly over multiple consultations or visits. Discrepancies may arise if there are mistakes while entering the data or if the patient chooses a different ethnic group when asked by the service provider [1]. The linked hospital data provided by CPRD (HES), contains one ethnicity grouping record for each patient; this variable is derived by CPRD and is the most frequently recorded ethnicity value across episodes and hospitalisations in Admitted Patient Care (APC), Outpatient and A&E data [2]. This process has not been carried out in the primary care data, with multiple records and discrepancies potentially occurring.

All ethnicity codes within CPRD and HES were collapsed into the five higher-levels groups developed by the ONS; White, Mixed, Asian/Asian British, Black/Black British, Other [3]. The data from the two sources was then combined, as shown in ESM Figure 1. If patients had no ethnic codes in CPRD, the HES ethnic group was assigned to the patient, if available. In patients with multiple ethnic codes recorded in CPRD, the ethnicity recorded in HES was used to clarify classification.

There were three types of multiple codings within CPRD and between CPRD and HES [1];

1. truly matched
   multiple ethnic codes which are identical
2. categorically matched
   multiple ethnic codes which are different but fall within the same five higher-level groups of ethnicity
3. truly mismatched
   multiple ethnicities which span different higher level groups.

After combining the data sources and generating a final ethnic classification defined into one of the five higher-level groups, the Asian/British Asian group was delineated into South Asians (Indian, Pakistani, Bangladeshi), with the remaining Asian/British Asian types (Chinese, other Asian) being re-classified as "other" (which includes East/Southeast Asian ethnicities; for example, Korean, Japanese, Vietnamese, etc.)

References

1. Mathur R, Bhaskaran K, Chaturvedi N, et al (2014) Completeness and usability of ethnicity data in UK-based primary care and hospital databases. J Public Health (Bangkok) 36(4):684–692
2. Medicines and Healthcare Products Regulatory Agency, CPRD (2019) Hospital Episode Statistics (HES) Admitted Patient Care and CPRD primary care data Documentation (Set 17). https://cprd.com/sites/default/files/HES APC documentation.pdf. Accessed 20 May 2019
3. Office for National Statistics (2015) Harmonised Concepts and Questions for Social Data Sources: Primary Principles. Ethnic Group. https://www.ons.gov.uk/ons/guide-method/harmonisation/primary-set-of-harmonised-concepts-and-questions/ethnic-group.pdf. Accessed 10 Sep 2016

Missing data.

Key differences in missingness

Missingness ranged between ~12-30% in people with type 2 diabetes and 36~70% in controls without diabetes (**ESM Table 1**).

People with missing data were more likely to be female with fewer prescriptions for antidiabetic medications, antihypertensives, lipid-lowering agents and antiplatelets (**ESM Table 2**). The lower rates of drug prescriptions in those with missing data may be related to the greater proportion of females in this group; it has shown previously that women tend to receive less treatment (initiation and intensification) for many modifiable risk factors.[4, 5] Missing data and lower prescribing may also be related to healthcare utilisation;[6] each interaction with the health care system and health care professionals provides an opportunity for documentation and implementation and modification of treatment interventions.

It is important to note that there were no clinically meaningful differences in biological factor measurements between those with and without any missing data within type 2 diabetes and control groups across the full cohort or when stratified by sex or ethnicity (**ESM Tables 2-4**).

References

4. Wright AK, Kontopantelis E, Emsley R, et al (2019) Cardiovascular Risk and Risk Factor Management in Type 2 Diabetes Mellitus: A Population-Based Cohort Study Assessing Sex Disparities. Circulation 139(24):2742–2753
5. Wexler D, Grant R, Meigs J, Nathan D, Cagliero E (2005) Sex disparities in treatment of cardiac risk factors in patients with type 2 diabetes. Diabetes Care 28(3):514–520
6. Wells BJ, Nowacki AS, Chagin K, Kattan MW (2013) Strategies for Handling Missing Data in Electronic Health Record Derived Data. eGEMs (Generating Evid Methods to Improv patient outcomes) 1(3):7

Imputation model

Variables included in the imputation model were: BMI, weight, height, blood pressure (systolic and diastolic), total cholesterol, HDL cholesterol, non-HDL cholesterol, triacylglycerol, sex, age, ethnicity, deprivation, calendar year, general practice, cardiovascular disease, renal disease, and drug prescriptions for antidiabetic medications, antihypertensive agents, lipid-lowering medications and antiplatelet agents.

ESM Table 1. Proportion of people with type 2 diabetes and controls with missing data on risk factors by sex and ethnicity

	All	Men	Women	White	South Asian	Black
**Type 2 diabetes, N**	187,601	103,607	83,994	143,481	9,486	4,451
**Controls, N**	906,182	495,778	410,404	652,388	13,917	10,532
**BMI**, kg/m^2						
Type 2 diabetes	18.6	17.7	19.6	17.9	23.3	24.0
Controls	63.9	66.9	60.3	59.4	59.9	59.5
**Weight**, kg						
Type 2 diabetes	17.0	16.2	17.9	16.4	21.4	22.4
Controls	62.9	66.0	59.1	58.3	59.0	58.6
**Total cholesterol**, mmol/l						
Type 2 diabetes	16.8	15.9	18.0	16.2	20.6	22.4
Controls	62.4	62.5	62.2	57.6	55.5	60.5
**HDL cholesterol**, mmol/l						
Type 2 diabetes	28.9	28.1	29.9	28.6	28.8	30.6
Controls	68.9	67.0	68.9	65.0	59.8	63.9
**non-HDL cholesterol**, mmol/l						
Type 2 diabetes	29.0	28.2	29.9	28.6	28.9	30.6
Controls	69.0	69.0	68.9	65.0	59.9	63.9
**Triacylglycerol**, mmol/l						
Type 2 diabetes	29.9	28.8	31.3	29.9	28.4	29.3
Controls	70.7	70.7	70.8	67.2	60.4	64.3
**Blood pressure**, mmHg						
Type 2 diabetes	12.5	12.4	12.7	11.9	18.1	18.3
Controls	36.4	42.2	29.4	30.1	36.6	37.6

ESM Table 2. Differences in clinical characteristics between those with and without missing data on biological risk factors

	Type 2 diabetes N=187,601			Controls without diabetes N=906,182		
	Without missing data	With missing data*	P-value	Without missing data	With missing data*	P-value
**N (%)**	115,183 (61.4)	72,418 (38.6)		152,023 (16.8)	754,159 (83.2)	
**Men**	64,752 (56.2)	38,855 (53.7)	<0.001	83,139 (54.7)	412,639 (54.7)	0.849
**Age, years**	61.5±13.2	62.6±15.4	<0.001	66.0±11.2	60.7±14.6	<0.001
**Ethnicity**			<0.001			<0.001
White	88,382 (76.7)	55,099 (76.1)		125,949 (82.9)	526,439 (69.8)	
South Asian	5,996 (5.2)	3,490 (4.8)		3,126 (2.1)	10,791 (1.4)	
Black	2,768 (2.4)	1,683 (2.3)		2,318 (1.5)	8,214 (1.1)	
Other	1,581 (1.4)	899 (1.2)		1,332 (0.9)	6,949 (0.9)	
Unknown	16,456 (14.3)	11,247 (15.5)		19,298 (12.7)	201,766 (26.8)	
**Deprivation (IMD 2010)**			<0.001			<0.001
IMD 1 (least)	21,599 (18.8)	12,691 (17.5)		32,135 (21.1)	165,085 (21.9)	
IMD 2	24,416 (21.2)	16,518 (22.8)		34,190 (22.5)	176,196 (23.4)	
IMD 3	23,061 (20.0)	15,476 (21.4)		29,965 (19.7)	155,994 (20.7)	
IMD 4	24,260 (21.1)	14,868 (20.5)		30,161 (19.8)	142,993 (19.0)	
IMD 5 (most)	21,678 (18.8)	12,701 (17.5)		25,402 (16.7)	112,838 (15.0)	
Unknown	169 (0.2)	164 (0.2)		170 (0.1)	1,053 (0.1)	
**Cardiovascular disease**	22,046 (19.1)	15,719 (21.7)	<0.001	24,687 (16.2)	43,747 (5.8)	<0.001
**Renal disease (CKD stage≥4)**	1,151 (1.0)	1,184 (1.6)	<0.001	531 (0.4)	1,850 (0.3)	<0.001
**Biological factors (±12 months from diagnosis)**						
**BMI**	31.5±6.8	30.9±7.1	<0.001	27.7±5.4	26.9±5.6	<0.001
**Weight**	89.4±20.8	87.3±21.6	<0.001	78.6±17.3	75.5±17.8	<0.001
**Total cholesterol**	5.2±1.3	5.1±1.3	<0.001	5.2±1.2	5.3±1.1	<0.001
**HDL-cholesterol**	1.2±0.4	1.2±0.4	0.079	1.5±0.4	1.5±0.4	0.063
**non-HDL cholesterol**	4.0±1.3	3.9±1.3	<0.001	3.7±1.1	3.8±1.1	<0.001

**Triacylglycerol**	2.3±2.4	2.5±3.7	<0.001	1.5±1.0	1.5±1.4	0.995
**Blood pressure**						
sBP	139±19	140±20	<0.001	137±18	137±29	0.230
dBP	81±11	81±12	0.059	79±10	80±10	<0.001
**Drug Prescriptions**						
**Diabetes**						
Any oral hypoglycaemic	57,125 (49.6)	25,564 (35.3)	<0.001	-	-	-
Metformin	47,749 (41.5)	17,081 (23.6)	<0.001	-	-	-
Sulphonylurea	11,608 (10.1)	9,056 (12.5)	<0.001	-	-	-
Insulin	3,647 (3.2)	3,041 (4.2)	<0.001	-	-	-
Other	2,229 (1.9)	853 (1.2)	<0.001	-	-	-
**Antihypertensive agent**						
Any	72,782 (63.2)	30,220 (41.7)	<0.001	89,995 (59.2)	183,781 (24.4)	<0.001
Alpha-blocker	5,733 (5.0)	2,307 (3.2)	<0.001	6,867 (4.5)	13,520 (1.8)	<0.001
Angiotensin-2 receptor blocker	12,444 (10.8)	3,889 (5.4)	<0.001	14,987 (9.9)	23,544 (3.1)	<0.001
ACE inhibitor	40,114 (34.8)	15,080 (20.8)	<0.001	39,358 (25.9)	65,867 (8.7)	<0.001
Beta-blocker	25,826 (22.4)	10,494 (14.5)	<0.001	32,602 (21.5)	60,306 (8.0)	<0.001
Calcium channel blocker	28,153 (24.4)	10,456 (14.4)	<0.001	35,071 (23.1)	61,844 (8.2)	<0.001
Diuretic: thiazide, potassium sparing or loop	34,971 (30.4)	16,572 (22.9)	<0.001	41,549 (27.3)	100,133 (13.3)	<0.001
**Lipid lowering therapy**						
Any	63,604 (55.2)	19,097 (26.4)	<0.001	63,723 (41.9)	76,540 (10.2)	<0.001
**Antiplatelets**						
Any	34,099 (29.6)	14,632 (20.2)	<0.001	42,811 (28.2)	72,155 (9.6)	<0.001

Differences in patient characteristics between those with any missing data and without missing data on biological risk factors were assessed using t-tests (continuous factors) and chi-square tests (categorical factors).
Cardiovascular disease included MI, stroke, CHD, PVD and angina

* Individuals included in the "with missing data" group had missing data on at least one of the biological factors; weight, blood pressure, total cholesterol, HDL cholesterol, non-HDL cholesterol, triacylglycerol

ESM Table 3. Differences in clinical characteristics between those with and without missing data on biological risk factors, by sex

	Men						Women					
	Type 2 diabetes N=103,607			Controls without diabetes N=495,778			Type 2 diabetes N=83,994			Controls without diabetes N=410,404		
	Without any missing data	With missing data*	P-value	Without any missing data	With missing data*	P-value	Without any missing data	With missing data*	P-value	Without any missing data	With missing data*	P-value
**N (%)**	64,752 (62.5)	38,855 (37.5)		83,139 (16.8)	412,639 (83.2)		50,431 (60.0)	33,563 (40.0)		68,884 (16.8)	341,520 (83.2)	
**Age, years**	60.4±12.7	60.5±14.5	0.080	65.1±10.9	58.9±13.7	<0.001	62.9±13.7	65.0±16.1	<0.001	67.0±11.6	62.9±15.3	<0.001
**Ethnicity**			<0.001			<0.001			<0.001			<0.001
White	76.2	75.2		82.7	66.6		77.4	77.1		83.1	73.7	
South Asian	5.0	4.9		1.9	1.4		5.5	4.7		2.2	1.5	
Black	2.1	2.1		1.2	1.0		2.8	2.5		1.9	1.2	
Other	1.4	1.3		0.8	0.9		1.4	1.2		11.9	1.0	
Unknown	15.3	16.5		13.3	30.1		12.9	14.5			22.7	
**Deprivation (IMD 2010)**			<0.001			<0.001			<0.001			<0.001
IMD 1 (least)	19.8	17.9		22.4	22.2		17.4	17.1		19.6	21.5	
IMD 2	21.7	23.5		23.1	23.4		20.6	22.0		21.8	23.4	
IMD 3	20.3	21.4		19.8	20.6		19.7	21.3		19.6	20.8	
IMD 4	20.4	20.3		19.0	18.9		21.9	20.8		20.8	19.1	
IMD 5 (most)	17.7	16.7		15.6	14.9		20.3	18.5		18.1	15.1	
Unknown	0.1	0.2		0.1	0.1		0.2	0.3		0.1	0.1	
**Cardiovascular disease**	21.9	23.8	<0.001	19.6	6.3	<0.001	15.6	19.3	<0.001	12.2	5.2	<0.001
**Renal disease (CKD stage≥4)**	1.0	1.6	<0.001	0.4	0.3	<0.001	1.0	1.7	<0.001	0.3	0.3	<0.001
**Biological factors (±12 months from diagnosis)**												
**BMI**	31.2±6.2	30.9±6.5	<0.001	27.9±4.8	27.4±5.2	<0.001	31.8±7.5	31.0±7.8	<0.001	27.5±5.9	26.5±6.0	<0.001
**Weight**	94.5±19.7	92.7±20.4	<0.001	84.7±15.8	83.0±16.7	<0.001	83.0±20.4	80.9±21.3	<0.001	71.1±15.9	69.1±16.3	<0.001
**Total cholesterol**	5.1±1.3	5.0±1.3	<0.001	4.9±1.1	5.1±1.1	<0.001	5.4±1.3	5.3±1.3	<0.001	5.5±1.1	5.5±1.1	<0.001

**HDL cholesterol**	1.1±0.3	1.1±0.4	0.384	1.3±0.4	1.3±0.4	<0.001	1.3±0.4	1.3±0.4	0.373	1.6±0.4	1.6±0.5	<0.001
**non-HDL cholesterol**	3.9±1.3	3.8±1.3	<0.001	3.6±1.1	3.8±1.1	<0.001	4.0±1.3	3.9±1.3	<0.001	3.8±1.1	3.9±1.1	<0.001
**Triacylglycerol**	2.4±2.8	2.7±4.1	<0.001	1.6±1.2	1.6±1.3	0.029	2.1±1.7	2.2±3.2	<0.001	1.4±0.8	1.4±1.5	0.197
**Blood pressure**												
sBP	139±18	140±19	<0.001	137±17	138±36	<0.001	139±20	141±21	<0.001	137±18	136±20	<0.001
dBP	82±11	81±11	<0.001	80±11	80±11	<0.001	80±11	80±12	<0.001	79±10	79±10	0.067
**Drug Prescriptions**												
**Diabetes**												
Any oral hypoglycaemic	50.4	35.1	<0.001	-	-	-	48.5	35.6	<0.001	-	-	-
Metformin	42.1	23.5	<0.001	-	-	-	40.7	23.7	<0.001	-	-	-
Sulphonylurea	10.6	12.4	<0.001	-	-	-	9.5	12.6	<0.001	-	-	-
Insulin	3.2	4.2	<0.001	-	-	-	3.2	4.2	<0.001	-	-	-
Other	1.9	1.2	<0.001	-	-	-	2.0	1.2	<0.001	-	-	-
**Antihypertensive agent**												
Any	61.2	39.5	<0.001	59.2	21.3	<0.001	65.8	44.3	<0.001	59.2	28.1	<0.001
Alpha-blocker	5.1	3.5	<0.001	5.2	2.0	<0.001	4.8	2.9	<0.001	3.7	1.5	<0.001
Angiotensin-2 receptor blocker	9.5	4.7	<0.001	8.9	2.6	<0.001	12.5	6.1	<0.001	11.1	3.8	<0.001
ACE inhibitor	36.9	21.8	<0.001	29.1	8.8	<0.001	32.2	19.8	<0.001	22.0	8.7	<0.001
Beta-blocker	22.5	14.5	<0.001	22.9	7.3	<0.001	22.4	14.5	<0.001	19.8	8.9	<0.001
Calcium channel blocker	24.3	14.1	<0.001	23.7	7.8	<0.001	24.7	14.8	<0.001	22.3	8.7	<0.001
Diuretic: thiazide, potassium sparing or loop	24.5	18.4	<0.001	22.7	9.6	<0.001	37.9	28.1	<0.001	33.0	17.7	<0.001
**Lipid lowering therapy**												
Any	56.8	27.9	<0.001	46.1	10.9	<0.001	53.2	24.6	<0.001	36.9	9.3	<0.001
**Antiplatelets**												
Any	31.7	20.9	<0.001	31.7	9.4	<0.001	26.9	19.5	<0.001	23.9	9.8	<0.001

Differences in patient characteristics between those with any missing data and without missing data on biological risk factors are presented as % and mean±SD (where appropriate) and assessed using t-tests (continuous factors) and chi-square tests (categorical factors).
Cardiovascular disease included MI, stroke, CHD, PVD and angina

* Individuals included in the “with missing data” group had missing data on at least one of the biological factors; weight, blood pressure, total cholesterol, HDL cholesterol, non-HDL cholesterol

ESM Table 4. Differences in clinical characteristics between those with and without missing data on biological risk factors, by ethnicity

	WHITE						SOUTH ASIAN					
	Type 2 diabetes N=143,481			Controls without diabetes N=652,388			Type 2 diabetes N=9,486			Controls without diabetes N=13,917		
	Without any missing data	With missing data*	P-value	Without any missing data	With missing data*	P-value	Without any missing data	With missing data*	P-value	Without any missing data	With missing data*	P-value
**N (%)**	88,382 (61.6)	55,099 (38.4)		125,949 (19.3)	526,439 (80.7)		5,996 (63.2)	3,490 (36.8)		3,126 (22.5)	10,791 (77.5)	
**Men**	55.8	53.1	<0.001	54.6	52.2	<0.001	54.0	54.6	0.516	51.0	53.6	0.010
**Age, years**	62.9±12.9	64.0±15.0	<0.001	66.8±11.0	62.3±14.4	<0.001	52.9±13.0	52.1±14.2	0.007	59.1±12.0	51.9±14.5	<0.001
**Deprivation (IMD 2010)**			<0.001			<0.001			<0.001			0.007
IMD 1 (least)	18.8	17.4		21.0	21.4		12.0	14.8		12.6	12.4	
IMD 2	22.1	23.8		23.0	24.1		14.4	15.5		17.6	15.7	
IMD 3	20.1	21.3		19.8	20.9		20.5	21.0		20.9	22.2	
IMD 4	20.7	20.2		19.6	18.7		25.4	22.3		23.6	25.9	
IMD 5 (most)	18.2	17.0		16.5	14.8		27.6	26.2		25.3	23.7	
Unknown	0.1	0.2		0.1	0.1		0.2	0.1		<0.1	0.1	
**Cardiovascular disease**	21.6	23.9	<0.001	17.3	6.7	<0.001	11.6	14.2	<0.001	10.5	4.1	<0.001
**Renal disease (CKD stage≥4)**	1.1	1.8	<0.001	0.4	0.3	<0.001	0.8	1.7	<0.001	0.7	0.5	0.13
**Biological factors (±12 months from diagnosis)**												
**BMI**	31.8±6.8	31.2±7.2	<0.001	27.7±5.4	26.9±5.7	<0.001	28.5±5.7	28.3±6.3	0.289	26.8±4.8	26.0±5.2	<0.001
**Weight**	90.5±20.9	88.1±21.8	<0.001	78.6±17.3	75.5±17.9	<0.001	77.6±16.1	76.8±16.9	0.071	71.5±14.3	68.9±14.7	<0.001
**Total cholesterol**	5.2±1.3	5.1±1.3	<0.001	5.2±1.2	5.2±1.2	<0.001	5.1±1.2	5.0±1.1	0.013	5.0±1.0	5.1±1.0	0.004
**HDL cholesterol**	1.2±0.4	1.2±0.4	0.226	1.5±0.4	1.5±0.4	<0.001	1.2±0.3	1.2±0.4	0.347	1.4±0.4	1.3±0.4	0.457
**non-HDL cholesterol**	3.9±1.3	3.8±1.3	<0.001	3.7±1.1	3.8±1.1	<0.001	3.9±1.2	3.9±1.2	0.573	3.7±1.0	3.8±1.0	<0.001
**Triacylglycerol**	2.3±2.3	2.5±3.3	<0.001	1.5±1.1	1.5±1.4	0.046	2.2±2.7	2.6±6.8	0.004	1.5±0.9	1.6±2.2	0.189
**Blood pressure**												
sBP	140±19	141±20	<0.001	137±18	137±31	0.369	133±18	135±19	<0.001	132±18	129±18	<0.001

dBP	81±11	80±11	<0.001	79±10	79±10	<0.001	81±10	81±11	0.974	79±10	78±10	0.002
**Drug Prescriptions**												
**Diabetes**												
Any oral hypoglycaemic	48.3	35.2	<0.001	-	-	-	57.8	30.1	<0.001	-	-	-
Metformin	40.1	23.4	<0.001	-	-	-	49.5	21.5	<0.001	-	-	-
Sulphonylurea	9.7	12.3	<0.001	-	-	-	12.5	11.0	0.025	-	-	-
Insulin	3.2	4.3	<0.001	-	-	-	3.2	3.2	0.950	-	-	-
Other	1.9	1.2	<0.001	-	-	-	2.6	1.5	<0.001	-	-	-
**Antihypertensive agent**												
Any	66.3	44.2	<0.001	60.7	28.2	<0.001	44.8	22.3	<0.001	44.9	15.9	<0.001
Alpha-blocker	5.3	3.4	<0.001	4.7	2.1	<0.001	2.9	1.3	<0.001	3.5	1.4	<0.001
Angiotensin-2 receptor blocker	11.2	5.6	<0.001	10.1	3.7	<0.001	9.8	4.9	<0.001	10.8	3.3	<0.001
ACE inhibitor	36.4	22.0	<0.001	26.9	10.2	<0.001	24.7	10.6	<0.001	18.0	5.5	<0.001
Beta-blocker	24.3	15.7	<0.001	22.3	9.4	<0.001	13.1	7.6	<0.001	15.5	5.2	<0.001
Calcium channel blocker	25.1	15.0	<0.001	23.3	9.5	<0.001	17.7	8.7	<0.001	20.4	7.0	<0.001
Diuretic: thiazide, potassium sparing or loop	32.9	24.8	<0.001	28.2	15.3	<0.001	14.4	7.5	<0.001	15.5	6.0	<0.001
**Lipid lowering therapy**												
Any	57.1	28.1	<0.001	43.9	12.1	<0.001	48.6	17.2	<0.001	32.9	8.8	<0.001
**Antiplatelets**												
Any	32.0	21.9	<0.001	30.0	11.5	<0.001	22.5	11.6	<0.001	20.4	6.1	<0.001

	**BLACK**					
	**Type 2 diabetes N=4,451**			**Controls without diabetes N=10,532**		
	**Without any missing data**	**With missing data***	**P-value**	**Without any missing data**	**With missing data***	**P-value**
**N (%)**	2,768 (62.2)	1,683 (37.8)		2,318 (22.0)	8,214 (78.0)	
**Men**	49.6	49.4	0.932	44.3	52.3	<0.001
**Age, years**	54.2±12.9	53.6±15.2	0.192	57.7±11.5	51.3±12.9	<0.001
**Deprivation (IMD 2010)**			<0.001			<0.001
IMD 1 (least)	4.6	5.7		3.1	4.6	

IMD 2	7.5	9.3		5.7	7.9	
IMD 3	14.6	18.4		11.5	15.5	
IMD 4	32.6	31.9		34.9	32.4	
IMD 5 (most)	40.6	34.4		44.7	39.5	
Unknown	0.1	0.2		0.2	0.2	
**Cardiovascular disease**	7.0	9.9	0.001	5.5	2.4	<0.001
**Renal disease (CKD stage≥4)**	1.0	1.6	0.083	0.5	0.6	0.452
**Biological factors (±12 months from diagnosis)**						
**BMI**	31.2±6.6	30.6±6.6	0.047	29.5±5.7	28.5±5.9	<0.001
**Weight**	88.3±18.9	85.5±19.4	0.001	82.2±16.2	79.9±16.9	<0.001
**Total cholesterol**	5.1±1.2	5.0±1.2	0.122	5.1±1.0	5.1±1.1	0.421
**HDL cholesterol**	1.3±0.4	1.3±0.5	0.105	1.5±0.4	1.5±0.4	0.006
**non-HDL cholesterol**	3.8±1.2	3.6±1.2	0.018	3.6±1.0	3.6±1.0	0.274
**Triacylglycerol**	1.5±1.5	1.5±1.4	0.100	1.1±0.7	1.1±0.5	0.737
**Blood pressure**						
sBP	137±18	139±20	0.004	136±18	134±27	0.001
dBP	83±11	82±11	0.274	82±11	81±11	<0.001
**Drug Prescriptions**						
**Diabetes**						
Any oral hypoglycaemic	62.9	32.9	<0.001	-	-	-
Metformin	52.4	22.0	<0.001	-	-	-
Sulphonylurea	14.6	11.4	0.002	-	-	-
Insulin	6.1	6.2	0.896	-	-	-
Other	2.6	1.0	<0.001	-	-	-
**Antihypertensive agent**						
Any	53.4	25.3	<0.001	49.5	17.6	<0.001
Alpha-blocker	6.1	3.4	<0.001	6.0	2.1	<0.001
Angiotensin-2 receptor blocker	9.5	4.3	<0.001	8.5	3.0	<0.001
ACE inhibitor	26.4	10.9	<0.001	14.5	4.9	<0.001
Beta-blocker	11.6	7.9	<0.001	10.3	4.4	<0.001

Calcium channel blocker	33.6	15.0	<0.001	33.6	11.1	<0.001
Diuretic: thiazide, potassium sparing or loop	24.1	12.0	<0.001	22.4	8.5	<0.001
**Lipid lowering therapy**						
Any	41.3	13.2	<0.001	19.8	5.5	<0.001
**Antiplatelets**						
Any	18.6	9.6	<0.001	11.7	3.7	<0.001

Differences in patient characteristics between those with any missing data and without missing data on biological risk factors are presented as % and mean±SD (where appropriate) and assessed using t-tests (continuous factors) and chi-square tests (categorical factors).
Cardiovascular disease included MI, stroke, CHD, PVD and angina.

* Individuals included in the “with missing data” group had missing data on at least one of the biological factors; weight, blood pressure, total cholesterol, HDL cholesterol, non-HDL cholesterol

ESM Table 5. Baseline characteristics of controls without diabetes

	All	Men	Women	White	South Asian	Black
**N**	906,182	495,778	410,404	652,388	13,917	10,532
**Men, n (%)**	495,778 (54.7)	-	-	343,347 (52.6)	7,377 (53.0)	5,324 (50.6)
**Age, years**	61.6 ± 14.2	59.9 ± 13.4	63.5 ± 14.8	63.2 ± 13.9	53.5 ± 14.3	52.7 ± 12.9
**Age group**						
20-39	59,989 (6.6)	33,895 (6.8)	26,094 (6.4)	34,749 (5.3)	2,518 (18.1)	1,496 (14.2)
40-49	128,893 (14.2)	79,785 (16.1)	49,108 (12.0)	78,113 (12.0)	3,209 (23.1)	3,232 (30.7)
50-59	206,230 (22.8)	125,128 (25.2)	81,102 (19.8)	137,838 (21.1)	3,430 (24.7)	2,787 (26.5)
60-69	231,378 (25.5)	131,432 (26.5)	99,946 (24.4)	174,795 (26.8)	2,606 (18.7)	1,702 (16.2)
70-79	180,942 (20.0)	88,160 (17.8)	92,782 (22.6)	145,731 (22.3)	1,668 (12.0)	1,074 (10.2)
≥80	98,750 (10.9)	37,378 (7.5)	61,372 (15.0)	81,162 (12.4)	486 (3.5)	241 (2.3)
**Ethnicity**						
White	652,388 (72.0)	343,347 (69.3)	309,041 (75.3)	-	-	-
South Asian	13,917 (1.5)	7,377 (1.5)	6,540 (1.6)	-	-	-
Black	10,532 (1.2)	5,324 (1.1)	5,208 (1.3)	-	-	-
Other	8,281 (0.9)	4,376 (1.0)	3,905 (1.0)	-	-	-
Unknown	221,064 (24.4)	135,354 (27.3)	85,710 (20.9)	-	-	-
**Deprivation (IMD 2010)**						
IMD 1 (least)	197,220 (21.8)	110,171 (22.2)	87,049 (21.2)	138,961 (21.3)	1,732 (12.5)	447 (4.2)
IMD 2	210,386 (23.2)	115,567 (23.3)	94,819 (23.1)	155,940 (23.9)	2,245 (16.1)	781 (7.4)
IMD 3	185,959 (20.5)	101,532 (20.5)	84,427 (20.6)	134,748 (20.7)	3,047 (21.9)	1,541 (14.6)
IMD 4	173,154 (19.1)	93,594 (18.9)	79,560 (19.4)	123,072 (18.9)	3,534 (25.4)	3,467 (32.9)
IMD 5 (most)	138,240 (15.3)	74,241 (15.0)	63,999 (15.6)	98,814 (15.2)	3,350 (24.1)	4,277 (40.6)
Unknown	1,223 (0.1)	673 (0.1)	550 (0.1)	853 (0.1)	9 (0.1)	19 (0.2)
**Cardiovascular disease**	68,434 (7.6)	42,280 (8.5)	26,154 (6.4)	57,015 (8.7)	765 (5.5)	322 (3.1)
**Renal disease (CKD stage≥4)**	2,381 (0.3)	1,320 (0.3)	1,061 (0.3)	1,942 (0.3)	70 (0.5)	61 (0.6)
**Biological factors (±12 months from diagnosis)**						
**BMI**, kg/m^2	27.3 ± 5.5	27.6 ± 5.0	26.9 ± 6.0	27.3 ± 5.5	26.5 ± 5.0	29.0 ± 5.8
missing (%)	*63.9*	*66.9*	*60.3*	*59.4*	*59.9*	*59.5*
**Weight**, kg	76.9 ± 17.7	83.8 ± 16.3	69.9 ± 16.2	77.0 ± 17.7	70.3 ± 14.6	81.1 ± 16.6
missing (%)	*62.9*	*66.0*	*59.1*	*58.3*	*59.0*	*58.6*
**Total cholesterol**, mmol/l	5.2 ± 1.2	5.0 ± 1.1	5.5 ± 1.1	5.2 ± 1.2	5.0 ± 1.0	5.1 ± 1.0
missing (%)	*62.4*	*62.5*	*62.2*	*57.6*	*55.5*	*60.5*
**HDL cholesterol**, mmol/l	1.5 ± 0.4	1.3 ± 0.4	1.6 ± 0.4	1.5 ± 0.4	1.3 ± 0.4	1.5 ± 0.4
missing (%)	*68.9*	*67.0*	*68.9*	*65.0*	*59.8*	*63.9*
**non-HDL cholesterol**, mmol/l	3.8 ± 1.1	3.7 ± 1.1	3.9 ± 1.1	3.7 ± 1.1	3.7 ± 1.0	3.6 ± 1.0
missing (%)	*69.0*	*69.0*	*68.9*	*65.0*	*59.9*	*63.9*
**Triacylglycerol** , mmol/l	1.5 ± 1.2	1.6 ± 1.3	1.4 ± 1.2	1.5 ± 1.2	1.6 ± 1.6	1.1 ± 0.6
missing (%)	*70.7*	*70.7*	*70.8*	*67.2*	*60.4*	*64.3*
**Blood pressure**, mmHg						
sBP	137 ± 26	137 ± 32	137 ± 20	137 ± 28	131 ± 18	134 ± 24
dBP	79 ± 10	80 ± 11	79 ± 10	79 ± 10	79 ± 10	81 ± 11
missing (%)	*36.4*	*42.2*	*29.4*	*30.1*	*36.6*	*37.6*
**Drug Prescriptions**						

**Diabetes**						
Any oral hypoglycaemic	0	0	0	0	0	0
Metformin	0	0	0	0	0	0
Sulphonylurea	0	0	0	0	0	0
Insulin	0	0	0	0	0	0
Other	0	0	0	0	0	0
**Antihypertensive agent**						
Any	30.2	27.6	33.3	34.5	22.4	24.6
Alpha-blocker	2.3	2.6	1.9	2.6	1.8	3.0
Angiotensin-2 receptor blocker	4.3	3.7	5.0	4.9	5.0	4.2
ACE inhibitor	11.6	12.2	10.9	13.4	8.3	7.0
Beta-blocker	10.3	9.9	10.7	11.9	7.5	5.7
Calcium channel blocker	10.7	10.5	11.0	12.1	10.0	16.0
Diuretic: thiazide, potassium sparing or loop	15.6	11.8	20.2	17.8	8.1	11.6
**Lipid lowering therapy**						
Any	15.5	16.8	13.9	18.3	14.2	8.6
**Antiplatelets**						
Any	12.7	13.1	12.2	15.1	9.3	5.5

Data are mean±SD, n(%) or %, where appropriate

Cardiovascular disease included MI, stroke, CHD, PVD and angina

ESM Table 6. Adjusted age-specific mean (95% CI) differences in biological risk factor levels in people with newly diagnosed type 2 diabetes compared to matched people without diabetes

Biological risk factor	Age	T2DM	Non-T2DM	Adjusted mean difference	P-value
**BMI** (kg/m^2)	**20-39**	34.6 ± 8.8	27.9 ± 7.4	6.7 (6.6,6.8)	<0.001
	**40-49**	33.8 ± 8.2	28.0 ± 7.5	5.8 (5.7,5.9)	<0.001
	50-59	32.6 ± 7.4	27.7 ± 5.7	4.9 (4.8,5.0)	*<0.001*
	**60-69**	31.0 ± 6.5	27.2 ± 5.6	3.8 (3.7,3.9)	<0.001
	**70-79**	29.2 ± 6.2	26.2 ± 6.6	2.9 (2.8,3.0)	<0.001
	**≥80**	26.6 ± 6.1	24.8 ± 6.1	1.8 (1.7,1.9)	<0.001
**Weight** (m)	**20-39**	99.7 ± 27.3	81.0 ± 22.5	18.7 (18.3,19.1)	<0.001
	**40-49**	97.9 ± 23.8	81.6 ± 27.3	16.4 (16.0,16.7)	<0.001
	**50-59**	93.7 ± 21.5	79.9 ± 18.9	13.8 (13.7,14.0)	<0.001
	**60-69**	88.2 ± 19.8	77.4 ± 16.9	10.7(10.6,10.9)	<0.001
	**70-79**	81.2 ± 18.6	73.2 ± 20.5	8.1 (7.8,8.3)	<0.001
	**≥80**	72.1 ± 17.0	66.9 ± 18.5	5.3 (5.0,5.5)	<0.001
**Systolic blood pressure** (mmHg)	**20-39**	131 ± 17	124 ± 19	7 (6,7)	<0.001
	**40-49**	135 ± 20	129 ± 24	6 (5,6)	<0.001
	**50-59**	139 ± 22	134 ± 20	5 (4,5)	<0.001
	**60-69**	141 ± 19	138 ± 36	3 (3,4)	<0.001
	**70-79**	142 ± 20	141 ± 20	1 (0.8,1.2)	<0.001
	**≥80**	141 ± 22	141 ± 22	-0.5 (-0.9,-0.2)	0.055
**Total cholesterol** (mmol/l)	**20-39**	5.5 ± 2.1	5.7 ± 2.3	-0.2 (-0.2,-0.1)	<0.001
	**40-49**	5.5 ± 1.4	5.6 ± 2.2	-0.1 (-0.1,0)	<0.001
	**50-59**	5.4 ± 1.3	5.6 ± 1.5	-0.2 (-0.2,-0.1)	<0.001
	**60-69**	5.2 ± 1.3	5.5 ± 1.5	-0.3 (-0.3,-0.2)	<0.001
	**70-79**	5.0 ± 1.4	5.3 ± 1.4	-0.3 (-0.4,-0.3)	<0.001
	**≥80**	4.9 ± 1.4	5.3 ± 1.6	-0.3 (-0.3,-0.2)	<0.001
**HDL cholesterol** (mmol/l)	**20-39**	1.1 ± 0.4	1.4 ± 0.4	-0.3 (-0.3,-0.2)	<0.001
	**40-49**	1.1 ± 0.4	1.4 ± 0.5	-0.3 (-0.3,-0.2)	<0.001
	**50-59**	1.2 ± 0.4	1.4 ± 0.5	-0.3 (-0.3,-0.2)	<0.001
	**60-69**	1.2 ± 0.4	1.5 ± 0.6	-0.2 (-0.3,-0.2)	<0.001
	**70-79**	1.3 ± 0.4	1.5 ± 1.0	-0.2 (-0.2,-0.1)	<0.001
	**≥80**	1.4 ± 0.5	1.6 ± 0.6	-0.2 (-0.2,-0.1)	<0.001
**non-HDL cholesterol** (mmol/l)	**20-39**	4.4 ± 2.1	4.3 ± 2.4	0.1 (0.1,0.2)	<0.001
	**40-49**	4.4 ± 1.4	4.2 ± 2.2	0.2 (0.1,0.2)	<0.001
	**50-59**	4.2 ± 1.3	4.2 ± 1.6	0.1 (0,0.1)	<0.001
	**60-69**	3.9 ± 1.3	4.0 ± 1.3	-0.1 (-0.1,0)	<0.001
	**70-79**	3.7 ± 1.3	3.8 ± 1.4	-0.1 (-0.2,-0.1)	<0.001
	**≥80**	3.6 ± 1.4	3.7 ± 1.4	-0.1 (-0.1,0)	<0.001
**Triacylglycerol** (mmol/l)	**20-39**	3.0 ± 5.9	1.6 ± 2.3	1.4 (1.3,1.5)	<0.001
	**40-49**	2.8 ± 4.3	1.6 ± 2.5	1.2 (1.2,1.3)	<0.001
	**50-59**	2.5 ± 3.4	1.5 ± 1.9	1.0 (0.9,1.0)	<0.001
	**60-69**	2.2 ± 2.3	1.5 ± 1.4	0.7 (0.6,0.7)	<0.001
	**70-79**	1.9 ± 1.9	1.4 ± 1.4	0.5 (0.4,0.5)	<0.001
	**≥80**	1.7 ± 1.7	1.3 ± 1.2	0.4 (0.3,0.4)	<0.001

Data are mean (95% CI) differences in risk factor levels between people with type 2 diabetes and controls without diabetes (level in people with type 2 diabetes minus level in controls). Differences are adjusted for between group differences in age, sex, general practice and deprivation

ESM Table 7. Adjusted age-specific mean (95% CI) differences in biological risk factor levels in men and women newly diagnosed with type 2 diabetes compared with people without diabetes

Biological risk factors	Age	Men				Women			
		T2DM	Non-T2DM	Adjusted mean difference (95% CI)	P-value	T2DM	Non-T2DM	Adjusted mean difference (95% CI)	P-value
**BMI** (kg/m^2)	**20-39**	34.1 ± 8.5	28.9 ± 8.9	5.2 (5.0,5.3)	<0.001	35.3 ± 9.5	26.6 ± 6.7	8.6 (8.4,8.9)	<0.001
	**40-49**	33.1 ± 7.7	28.6 ± 7.6	4.5 (4.4,4.6)	<0.001	35.1 ± 8.7	27.1 ± 6.9	7.9 (7.7,8.0))	<0.001
	**50-59**	32.1 ± 6.7	28.1 ± 5.8	4.1 (4.0,4.1)	<0.001	33.3 ± 8.0	27.0 ± 7.2	6.2 (6.1,6.3)	<0.001
	**60-69**	30.8 ± 5.9	27.5 ± 5.9	3.3 (3.2,3.4)	<0.001	31.3 ± 7.1	26.7 ± 5.9	4.5 (4.4,4.6)	<0.001
	**70-79**	29.0 ± 5.4	26.6 ± 5.8	2.4 (2.3,2.5)	<0.001	29.3 ± 6.7	25.9 ± 6.2	3.4 (3.3,3.5)	<0.001
	**≥80**	27.1 ± 5.1	25.4 ± 5.5	1.7 (1.6,1.8)	<0.001	26.3 ± 6.2	24.4 ± 5.7	1.9 (1.8,2.0)	<0.001
**Weight** (m)	**20-39**	103.3 ± 27.5	88.2 ± 25.2	15.2 (14.6,15.7)	<0.001	95.0 ± 27.0	71.7 ± 18.8	23.1 (22.5,23.7)	<0.001
	**40-49**	100.7 ± 24.2	87.2 ± 25.3	13.6 (13.2,14.0)	<0.001	93.3 ± 24.0	72.5 ± 21.3	20.7 (20.3,21.1)	<0.001
	**50-59**	97.6 ± 20.9	85.3 ± 17.4	12.3 (12.1,12.6)	<0.001	87.7 ± 21.4	71.6 ± 19.7	16.0 (15.7,16.4)	<0.001
	**60-69**	92.7 ± 18.6	83.0 ± 17.0	9.8 (9.6,10.0)	<0.001	81.9 ± 19.1	70.1± 15.1	11.7 (11.5,12.0)	<0.001
	**70-79**	86.5 ± 17.2	79.6 ± 18.2	7.0 (6.7,7.3)	<0.001	75.9 ± 17.2	67.1 ± 16.9	8.7 (8.5,9.0)	<0.001
	**≥80**	79.6 ± 15.0	74.7 ± 18.7	4.9 (4.5,5.3)	<0.001	67.4 ± 15.4	62.1 ± 15.7	5.3 (5.0,5.6)	<0.001
**Systolic blood pressure** (mmHg)	**20-39**	133 ± 18	127 ± 23	6 (5,7)	<0.001	129 ± 17	121 ± 18	8 (7,9)	<0.001
	**40-49**	136 ± 18	131 ± 24	5 (4,5)	<0.001	135 ± 22	127 ± 24	8 (7,8)	<0.001
	**50-59**	139 ± 22	135 ± 18	4 (3,4)	<0.001	139 ± 21	132 ± 20	6 (6,7)	<0.001
	**60-69**	141 ± 20	138 ± 30	3 (2,3)	<0.001	142 ± 19	138 ± 22	4 (3,4)	<0.001
	**70-79**	141 ± 20	140 ± 25	1 (1,2)	<0.001	143 ± 21	142 ± 22	1 (1,2)	<0.001
	**≥80**	139 ± 21	140 ± 22	-1 (-1,0)	0.002	142 ± 22	143 ± 23	-0.3 (-0.7,0.1)	0.132
**Total cholesterol** (mmol/l)	**20-39**	5.5 ± 2.2	5.5 ± 1.9	0 (-0.1,0.1)	0.973	5.4 ± 1.5	5.9 ± 1.9	-0.4 (-0.5,-0.4)	<0.001
	**40-49**	5.5 ± 1.5	5.5 ± 1.6	0 (-0.1,0.1)	0.665	5.5 ± 1.3	5.8 ± 2.2	-0.3 (-0.3,-0.2)	<0.001
	**50-59**	5.3 ± 1.4	5.5 ± 1.3	-0.1 (-0.2,-0.1)	<0.001	5.6 ± 1.4	5.8 ± 1.5	-0.2 (-0.3,-0.2)	<0.001
	**60-69**	5.0 ± 1.3	5.3 ± 1.3	-0.3 (-0.3,-0.2)	<0.001	5.5 ± 1.4	5.8 ± 1.6	-0.3 (-0.3,-0.2)	<0.001
	**70-79**	4.7 ± 1.3	5.0 ± 1.2	-0.3 (-0.3,-0.2)	<0.001	5.3 ± 1.4	5.6 ± 1.5	-0.3 (-0.4,-0.3)	<0.001
	**≥80**	4.6 ± 1.3	4.9 ± 1.2	-0.3 (-0.3,-0.2)	<0.001	5.2 ± 1.5	5.5 ± 1.7	-0.3 (-0.3,-0.2)	<0.001
**HDL cholesterol** (mmol/l)	**20-39**	1.0 ± 0.3	1.3 ± 0.4	-0.2 (-0.3,-0.2)	<0.001	1.2 ± 0.4	1.6 ± 0.5	-0.4 (-0.4,-0.3)	<0.001
	**40-49**	1.1 ± 0.3	1.3 ± 0.4	-0.2 (-0.2,-0.1)	<0.001	1.2 ± 0.4	1.6 ± 0.7	-0.4 (-0.4,-0.3)	<0.001
	**50-59**	1.1 ± 0.3	1.3 ± 0.4	-0.2 (-0.2,-0.1)	<0.001	1.3 ± 0.4	1.6 ± 0.7	-0.3 (-0.4,-0.3)	<0.001

	**60-69**	1.2 ± 0.4	1.3 ± 0.6	-0.2 (-0.2,-0.1)	<0.001	1.3 ± 0.4	1.6 ± 0.6	-0.3 (-0.3,-0.2)	<0.001
	**70-79**	1.2 ± 0.4	1.4 ± 0.6	-0.2 (-0.2,-0.1)	<0.001	1.4 ± 0.4	1.7 ± 1.1	-0.3 (-0.3,-0.2)	<0.001
	**≥80**	1.2 ± 0.4	1.4 ± 0.5	-0.2 (-0.2,-0.1)	<0.001	1.5 ± 0.5	1.7 ± 0.6	-0.2 (-0.3,-0.2)	<0.001
**non-HDL cholesterol** (mmol/l)	**20-39**	4.5 ± 2.2	4.3 ± 2.0	0.3 (0.2,0.3)	<0.001	4.3 ± 1.5	4.3 ± 1.9	0 (-0.1,0.1)	0.705
	**40-49**	4.4 ± 1.4	4.2 ± 1.7	0.2 (0.2,0.3)	<0.001	4.3 ± 1.4	4.2 ± 2.3	0.1 (0.1,0.2)	<0.001
	**50-59**	4.2 ± 1.3	4.1 ± 1.4	0.1 (0,0.1)	<0.001	4.3 ± 1.5	4.2 ± 1.5	0.1 (0.1,0.2)	<0.001
	**60-69**	3.8 ± 1.4	3.9 ± 1.2	-0.1 (-0.1,0)	<0.001	4.1 ± 1.4	4.1 ± 1.5	-0.1 (-0.1,0)	0.024
	**70-79**	3.5 ± 1.3	3.7 ± 1.2	-0.1 (-0.2,-0.1)	<0.001	3.9 ± 1.4	4.0 ± 1.5	-0.1 (-0.1,0)	<0.001
	**≥80**	3.3 ± 1.3	3.5 ± 1.3	-0.1 (-0.2,-0.1)	<0.001	3.7 ± 1.5	3.8 ± 1.4	-0.1 (-0.2,-0.1)	<0.001
**Triacylglycerol** (mmol/l)	**20-39**	3.4 ± 5.9	1.6 ± 1.9	1.8 (1.7,1.9)	<0.001	2.5 ± 4.5	1.5 ± 2.0	1.0 (0.9,1.1)	<0.001
	**40-49**	3.1 ± 4.5	1.6 ± 2.5	1.4 (1.4,1.5)	<0.001	2.4 ± 3.1	1.4 ± 1.5	0.9 (0.9,1.0)	<0.001
	**50-59**	2.6 ± 3.7	1.6 ± 1.9	1.0 (1.0,1.1)	<0.001	2.3 ± 2.6	1.5 ± 1.7	0.8 (0.8,0.9)	<0.001
	**60-69**	2.2 ± 2.7	1.5 ± 1.6	0.7 (0.6,0.7)	<0.001	2.1 ± 2.5	1.5 ± 1.3	0.7 (0.6,0.7)	<0.001
	**70-79**	1.9 ± 1.7	1.4 ± 1.5	0.5 (0.4,0.5)	<0.001	1.9 ± 1.8	1.4 ± 1.0	0.5 (0.5,0.6)	<0.001
	**≥80**	1.7 ± 1.5	1.3 ± 0.9	0.4 (0.3,0.4)	<0.001	1.7 ± 1.7	1.3 ± 1.3	0.4 (0.3,0.4)	<0.001

Data are mean (95% CI) differences in risk factor levels between men with T2DM and men without diabetes, and between women with T2DM and women without diabetes (e.g. level in people with T2DM minus level in controls). Differences are adjusted for between group differences in age, sex, general practice and deprivation

ESM Table 8. Adjusted age-specific mean (95% CI) differences in biological risk factor levels in Whites, South Asians and Blacks newly diagnosed with type 2 diabetes compared with people without diabetes

Biological risk factors	Age	White				South Asian				Black			
		DM	Non-DM	Adjusted mean difference (95% CI)	P-value	DM	Non-DM	Adjusted mean difference (95% CI)	P-value	DM	Non-DM	Adjusted mean difference (95% CI)	P-value
**BMI** (kg/m^2)	**20-39**	36.3 ± 9.0	27.8 ± 7.0	8.4 (8.2,8.5)	<0.001	30.6 ± 7.9	26.6 ± 6.2	4.1 (3.7,4.5)	<0.001	32.9 ± 9.5	28.0 ± 7.4	4.9 (4.1,5.8)	<0.001
	**40-49**	35.0 ± 8.0	28.1 ± 7.4	6.8 (6.7,6.9)	<0.001	29.5 ± 6.7	26.9 ± 5.7	2.6 (2.3,2.9)	<0.001	32.0 ± 7.2	28.6 ± 7.2	3.4 (2.9,3.8)	<0.001
	**50-59**	33.2 ± 8.1	27.8 ± 6.2	5.4 (5.3,5.5)	<0.001	28.6 ± 5.7	26.8 ± 5.5	1.8 (1.5,2.1)	<0.001	31.4 ± 7.3	28.7 ± 6.6	2.7 (2.3,3.2)	<0.001
	**60-69**	31.4 ± 6.4	27.3 ± 5.6	4.1 (4.0,4.2)	<0.001	27.5 ± 5.7	26.4 ± 6.4	1.1 (0.8,1.5)	<0.001	29.6 ± 6.0	28.2 ± 5.8	1.3 (0.8,1.8)	<0.001
	**70-79**	29.3 ± 6.5	26.3 ± 7.1	3.0 (2.9,3.1)	<0.001	26.9 ± 5.9	25.5 ± 5.3	1.3 (0.9,1.8)	<0.001	29.0 ± 6.2	27.4 ± 6.3	1.6 (1.0,2.3)	<0.001
	**≥80**	26.7 ± 6.2	24.8 ± 6.5	1.9 (1.8,2.0)	<0.001	25.3 ± 5.8	24.1 ± 6.0	1.2 (0.2,2.2)	0.017	27.1 ± 6.3	25.7 ± 5.6	1.4 (0.2,2.6)	0.025
**Weight** (m)	**20-39**	104.8 ± 26.6	80.4 ± 23.1	24.3 (23.8,24.9)	<0.001	85.8 ± 24.2	74.7 ± 19.9	11.1 (9.8,12.3)	<0.001	94.2 ± 25.6	80.0 ± 21.6	14.2 (11.9,16.4)	<0.001
	**40-49**	101.6 ± 23.7	81.8 ± 25.9	19.8 (19.4,20.2)	<0.001	81.8 ± 19.7	75.2 ± 17.3	6.6 (5.6,7.7)	<0.001	92.0 ± 21.0	82.1 ± 19.4	9.8 (8.5,11.1)	<0.001
	**50-59**	95.9 ± 22.8	80.3 ± 19.6	15.6 (15.4,15.9)	<0.001	78.4 ± 16.6	73.7 ± 16.6	4.8 (3.9,5.6)	<0.001	88.4 ± 19.9	81.5 ± 18.4	6.9 (5.6,8.2)	<0.001
	**60-69**	89.4 ± 19.8	77.8 ± 17.2	11.6 (11.4,11.8)	<0.001	74.4 ± 15.2	71.4 ± 18.0	2.9 (2.0,3.8)	<0.001	82.3 ± 16.4	78.5 ± 15.5	3.9 (2.5,5.2)	<0.001
	**70-79**	81.8 ± 19.3	73.5 ± 23.3	8.3 (8.1,8.6)	<0.001	71.7 ± 15.6	68.0 ± 14.6	3.8 (2.6,5.0)	<0.001	79.4 ± 15.2	75.1 ± 17.3	4.3 (2.6,6.1)	<0.001
	**≥80**	72.5 ± 16.5	67.1 ± 19.0	5.4 (5.2,5.7)	<0.001	66.3 ± 17.0	63.5 ± 16.1	2.9 (0.1,5.8)	<0.001	73.5 ± 16.8	70.4 ± 16.3	3.2 (0.2,6.5)	<0.001
**Systolic blood pressure** (mmHg)	**20-39**	132 ± 17	124 ± 17	9 (8,9)	<0.001	127 ± 17	121 ± 21	7 (5,8)	<0.001	130 ± 20	124 ± 19	7 (5,8)	<0.001
	**40-49**	136 ± 21	129 ± 22	7 (7,8)	<0.001	131 ± 20	126 ± 21	5 (4,6)	<0.001	134 ± 19	130 ± 22	4 (2,6)	<0.001
	**50-59**	139 ± 19	134 ± 19	5 (5,6)	<0.001	135 ± 23	131 ± 20	4 (3,5)	<0.001	138 ± 21	134 ± 22	5 (3,6)	<0.001
	**60-69**	141 ± 21	138 ± 24	3 (3,4)	<0.001	139 ± 19	136 ± 17	3 (2,4)	<0.001	143 ± 21	139 ± 21	5 (3,6)	<0.001
	**70-79**	142 ± 20	141 ± 20	1 (1,2)	<0.001	140 ± 21	138 ± 19	2 (1,3)	0.025	143 ± 21	141 ± 22	2 (-1,4)	0.056
	**≥80**	141 ± 22	141 ± 21	-0.4 (-0.7,0.1)	0.053	140 ± 22	139 ± 21	2 (-2,6)	0.319	140 ± 22	143 ± 23	-3 (-7,2)	0.260
**Total cholesterol** (mmol/l)	**20-39**	5.5 ± 1.6	5.7 ± 2.1	-0.2 (-0.2,-0.1)	<0.001	5.3 ± 1.6	5.5 ± 1.5	-0.2 (-0.3,-0.1)	0.001	5.3 ± 1.8	5.6 ± 1.6	-0.2 (-0.4,-0.1)	0.024
	**40-49**	5.5 ± 1.5	5.6 ± 1.9	-0.1 (-0.1,0)	<0.001	5.4 ± 1.3	5.5 ± 1.3	-0.1 (-0.2,-0.1)	0.002	5.3 ± 1.3	5.4 ± 1.3	-0.1 (-0.2,-0.1)	0.008
	**50-59**	5.4 ± 1.4	5.6 ± 1.5	-0.2 (-0.2,-0.1)	<0.001	5.2 ± 1.5	5.4 ± 1.1	-0.2 (-0.3,-0.1)	<0.001	5.3 ± 1.3	5.4 ± 1.4	-0.1 (-0.2,-0.1)	0.007
	**60-69**	5.2 ± 1.4	5.5 ± 1.4	-0.3 (-0.3,-0.2)	<0.001	5.1 ± 1.3	5.2 ± 1.2	-0.2 (-0.3,-0.1)	<0.001	5.2 ± 1.3	5.4 ± 1.2	-0.3 (-0.4,-0.2)	<0.001
	**70-79**	5.0 ± 1.4	5.3 ± 1.3	-0.3 (-0.3,-0.2)	<0.001	4.8 ± 1.4	5.0 ± 1.2	-0.2 (-0.3,-0.1)	<0.001	5.0 ± 1.3	5.2 ± 1.3	-0.1 (-0.3,0)	0.037
	**≥80**	4.9 ± 1.5	5.2 ± 1.4	-0.3 (-0.3,-0.2)	<0.001	4.8 ± 1.2	5.0 ± 1.3	-0.2 (-0.4,0)	0.057	4.8 ± 1.3	5.2 ± 1.4	-0.4 (-0.6,-0.1)	0.016
**HDL cholesterol**	**20-39**	1.1 ± 0.4	1.4 ± 0.5	-0.3 (-0.4,-0.3)	<0.001	1.1 ± 0.3	1.4 ± 0.4	-0.3 (-0.4,-0.3)	<0.001	1.2 ± 0.4	1.4± 0.5	-0.3 (-0.3,-0.2)	<0.001
	**40-49**	1.1 ± 0.4	1.4 ± 0.5	-0.3 (-0.3,-0.2)	<0.001	1.1 ± 0.4	1.4 ± 0.6	-0.3 (-0.3,-0.2)	<0.001	1.2 ± 0.4	1.4 ± 0.5	-0.2 (-0.2,-0.1)	<0.001

(mmol/l)	**50-59**	1.2 ± 0.4	1.4 ± 0.5	-0.3 (-0.3,-0.2)	<0.001	1.2 ± 0.3	1.4 ± 0.5	-0.2 (-0.2,-0.1)	<0.001	1.3 ± 0.4	1.5 ± 0.5	-0.2 (-0.2,-0.1)	<0.001
	**60-69**	1.2 ± 0.4	1.5 ± 0.7	-0.2 (-0.2,-0.1)	<0.001	1.2 ± 0.4	1.4 ± 0.5	-0.2 (-0.2,-0.1)	<0.001	1.3 ± 0.4	1.5 ± 0.5	-0.2 (-0.2,-0.1)	<0.001
	**70-79**	1.3 ± 0.4	1.5 ± 0.9	-0.2 (-0.2,-0.1)	<0.001	1.3 ± 0.4	1.5 ± 0.5	-0.2 (-0.2,-0.1)	<0.001	1.4 ± 0.5	1.5 ± 0.5	-0.1 (-0.2,0)	0.002
	**≥80**	1.4 ± 0.5	1.6 ± 0.6	-0.2 (-0.2,-0.1)	<0.001	1.3 ± 0.4	1.5 ± 0.5	-0.2 (-0.3,-0.1)	<0.001	1.4 ± 0.4	1.6 ± 0.5	-0.1 (-0.2,-0.1)	0.007
**non-HDL cholesterol** (mmol/l)	**20-39**	4.5 ± 1.8	4.3 ± 2.3	0.2 (0.1,0.2)	<0.001	4.3 ± 1.6	4.1 ± 1.7	0.2 (0.1,0.3)	0.005	4.2 ± 1.7	4.1 ± 1.6	0.1 (-0.1,0.2)	0.633
	**40-49**	4.4 ± 1.5	4.2 ± 1.9	0.2 (0.2,0.3)	<0.001	4.2 ± 1.3	4.1 ± 1.2	0.2 (0.1,0.2)	<0.001	4.1 ± 1.3	4.0 ± 1.5	0.1 (-0.1,0.2)	0.113
	**50-59**	4.3 ± 1.4	4.2 ± 1.6	0.1 (0.1,0.2)	<0.001	4.0 ± 1.4	4.0 ± 1.1	0 (-0.1,0.1)	0.473	4.0 ± 1.4	3.9 ± 1.3	0.1 (-0.1,0.1)	0.282
	**60-69**	3.9 ± 1.4	4.0 ± 1.4	-0.1 (-0.1,0)	<0.001	3.8 ± 1.2	3.8 ± 1.2	0 (-0.1,0.1)	0.819	3.8 ± 1.3	3.9 ± 1.2	-0.1 (-0.2,0.1)	0.068
	**70-79**	3.7 ± 1.3	3.8 ± 1.2	-0.1 (-0.1,0)	<0.001	3.6 ± 1.4	3.6 ± 1.3	0 (-0.1,0.1)	0.991	3.6 ± 1.3	3.6 ± 1.2	0 (-0.2,0.1)	0.562
	**≥80**	3.6 ± 1.5	3.6 ± 1.3	-0.1 (-0.1,0)	<0.001	3.5 ± 1.1	3.4 ± 1.2	0 (-0.1,0.2)	0.930	3.4 ± 1.3	3.6 ± 1.4	-0.2 (-0.5,0.1)	0.164
**Triacylglycerol** (mmol/l)	**20-39**	3.2 ± 5.3	1.6 ± 2.9	1.6 (1.5,1.7)	<0.001	2.8 ± 6.5	1.5 ± 1.0	1.3 (1.0,1.6)	<0.001	2.1 ± 2.9	1.5 ± 2.1	0.6 (0.4,0.9)	<0.001
	**40-49**	3.0 ± 4.5	1.6 ± 2.3	1.4 (1.3,1.5)	<0.001	2.4 ± 2.2	1.5 ± 1.3	0.9 (0.8,1.0)	<0.001	1.9 ± 2.4	1.4 ± 1.3	0.5 (0.3,0.6)	<0.001
	**50-59**	2.6 ± 3.3	1.6 ± 1.9	1.0 (0.9,1.0)	<0.001	2.2 ± 2.2	1.5 ± 1.1	0.6 (0.5,0.7)	<0.001	1.7 ± 2.3	1.4 ± 1.3	0.3 (0.2,0.5)	<0.001
	**60-69**	2.2 ± 2.2	1.5 ± 1.5	0.7 (0.6,0.7)	<0.001	2.0 ± 4.2	1.5 ± 2.2	0.5 (0.3,0.7)	<0.001	1.6 ± 1.6	1.3 ± 1.8	0.2 (0.1,0.4)	0.026
	**70-79**	1.9 ± 2.0	1.4 ± 1.3	0.5 (0.4,0.5)	<0.001	1.9 ± 2.3	1.4 ± 1.0	0.5 (0.3,0.6)	<0.001	1.4 ± 0.9	1.2 ± 1.3	0.1 (0,0.2)	0.046
	**≥80**	1.7 ± 1.5	1.3 ± 1.1	0.4 (0.3,0.4)	<0.001	1.7 ± 1.2	1.3 ± 0.9	0.4 (0.3,0.6)	<0.001	1.4 ± 1.4	1.2 ± 1.4	0.1 (-0.2,0.5)	0.363

Data are mean (95% CI) differences in risk factor levels between people T2DM and controls without diabetes, stratified by ethnic group (e.g. level in South Asians with T2DM minus level in South Asian controls). Differences are adjusted for between group differences in age, sex, general practice and deprivation

ESM Table 9. Adjusted mean (95%CI) differences in biological risk factor levels at age of diabetes onset between those newly diagnosed with type 2 diabetes compared with people without diabetes, by ethnicity and sex

Biological risk factors	Age	White		South Asian		Black	
		Women	Men	Women	Men	Women	Men
		Adjusted mean difference (95% CI)	Adjusted mean difference (95% CI)	Adjusted mean difference (95% CI)	Adjusted mean difference (95% CI)	Adjusted mean difference (95% CI)	Adjusted mean difference (95% CI)
**BMI** (kg/m^2)	**20-39**	10.2 (9.9,10.4) *	6.6 (6.3,6.8) *	5.3 (4.7,6.0) *	2.8 (2.3,3.4) *	5.9 (5.0,6.9) *	4.0 (2.8,5.2) *
	**40-49**	8.9 (8.8,9.1) *	5.4 (5.2,5.5) *	4.0 (3.5,4.6) *	1.5 (1.1,2.0) *	4.3 (3.6,5.0) *	2.7 (2.1,3.3) *
	**50-59**	6.8 (6.7,6.9) *	4.5 (4.4,4.6) *	2.4 (2.0,2.9) *	1.3 (0.9,1.7) *	3.1 (2.4,3.8) *	2.1 (1.5,2.7) *
	**60-69**	4.9 (4.8,5.0) *	3.5 (3.4,3.6) *	1.1 (0.6,1.7) *	1.1 (0.7,1.6) *	1.4 (0.6,2.1) *	1.3 (0.7,2.0) *
	**70-79**	3.5 (3.4,3.6) *	2.4 (2.3,2.5) *	1.5 (0.8,2.2) *	1.2 (0.7,1.7) *	1.5 (0.6,2.4) *	1.7 (0.8,2.5) *
	**≥80**	2.0 (1.9,2.1) *	1.7 (1.6,1.9) *	1.0 (-0.1,2.2)	1.4 (-0.1,2.8)	1.8 (0.1,3.5) *	0.9 (-0.7,2.6)
**Weight** (m)	**20-39**	27.6 (26.9,28.3) *	20.2 (19.5,20.9) *	13.1 (11.4,14.7) *	7.6 (5.7,9.4) *	16.6 (14.2,19.0) *	11.1 (7.6,14.7) *
	**40-49**	27.8 (23.3,24.2) *	16.6 (16.0,17.1) *	9.9 (8.6,11.3) *	3.3 (1.9,4.7) *	11.8 (10.0,13.6) *	7.8 (6.2,9.4) *
	**50-59**	17.7 (17.4,18.1) *	13.7 (13.4,14.0) *	6.7 (5.7,7.8) *	3.2 (2.0,4.3) *	8.3 (6.5,10.1) *	6.2 (4.4,8.0) *
	**60-69**	12.7 (12.4,13.0) *	10.3 (10.1,10.6) *	3.6 (2.5,4.8) *	2.9 (1.6,4.2) *	4.3 (2.3,6.3) *	3.3 (1.3,5.3) *
	**70-79**	9.0 (8.7,9.3) *	7.2 (6.8,7.6) *	4.6 (2.9,6.3) *	3.1 (1.5,4.8) *	4.4 (2.1,6.8) *	4.7 (2.3,7.1) *
	**≥80**	5.4 (5.1,5.7) *	5.1 (4.7,5.4) *	2.6 (-0.1,6.0)	3.8 (-0.4,7.9)	5.4 (0.8,10.0) *	1.3 (-3.1,5.7)
**Systolic blood pressure** (mmHg)	**20-39**	9.7 (9.0,10.3) *	7.6 (6.9,8.3) *	7.6 (5.7,9.5) *	5.2 (3.7,6.7) *	6.8 (4.0,9.6) *	6.1 (3.6,8.6) *
	**40-49**	8.9 (8.4,9.4) *	6.0 (5.6,6.4) *	6.9 (5.0,8.8) *	3.0 (1.8,4.3) *	4.1 (1.9,6.4) *	3.7 (0.8,6.5) *
	**50-59**	6.8 (6.4,7.2) *	4.4 (4.1,4.7) *	5.0 (3.6,6.4) *	3.2 (1.6,4.8) *	4.9 (3.0,6.7) *	4.5 (2.5,6.5) *
	**60-69**	4.1 (3.8,4.5) *	2.6 (2.0,3.3) *	3.7 (2.1,5.2) *	2.1 (0.5,3.6) *	5.2 (3.0,7.4) *	3.6 (1.2,5.9) *
	**70-79**	1.3 (1.0,1.6) *	1.2 (0.8,1.5) *	2.4 (0.1,4.7) *	1.4 (-0.9,3.6)	0.9 (-2.3,4.1)	3.5 (0.4,6.7) *
	**≥80**	-0.2 (-0.7,0.2)	-0.5 (-1.0,0.1)	2.5 (-2.1,7.2)	1.0 (-5.0,7.0)	-2.6 (-8.9,3.6)	-2.5 (-8.7,3.8)
**Total cholesterol** (mmol/l)	**20-39**	-0.4 (-0.4,-0.3) *	0.1 (-0.1,0.1)	-0.4 (-0.5,-0.3) *	-0.1 (-0.1,0.1)	-0.4 (-0.6,-0.2) *	-0.1 (-0.4,0.2)
	**40-49**	-0.2 (-0.3,-0.2) *	0.1 (0.1,0.2) *	-0.2 (-0.3,-0.1) *	-0.1 (-0.1,0.1)	-0.2 (-0.4,-0.1) *	-0.1 (-0.1,0.1)
	**50-59**	-0.2 (-0.2,-0.1) *	-0.1 (-0.2,-0.1) *	-0.2 (-0.3,-0.1) *	-0.2 (-0.3,-0.1) *	-0.1 (-0.3,0.1)	-0.2 (-0.3,-0.1) *
	**60-69**	-0.3 (-0.3,-0.2) *	-0.3 (-0.3,-0.2) *	-0.2 (-0.3,-0.1) *	-0.2 (-0.3,-0.1) *	-0.3 (-0.4,-0.1) *	-0.2 (-0.4,-0.1) *
	**70-79**	-0.3 (-0.4,-0.3) *	-0.3 (-0.3,-0.2) *	-0.2 (-0.3,0.1)	-0.3 (-0.4,-0.1) *	-0.2 (-0.4,-0.1) *	-0.1 (-0.3,0.1)
	**≥80**	-0.3 (-0.3,-0.2) *	-0.3 (-0.3,-0.2) *	-0.3 (-0.5,0.1)	-0.2 (-0.5,0.2)	-0.5 (-1.0,-0.1) *	-0.2 (-0.6,0.2)
	**20-39**	-0.4 (-0.4,-0.3) *	-0.2 (-0.3,-0.2) *	-0.4 (-0.4,-0.3) *	-0.2 (-0.3,-0.2) *	-0.3 (-0.4,-0.2) *	-0.2 (-0.3,-0.1) *

**HDL cholesterol** (mmol/l)	**40-49**	-0.4 (-0.4,-0.3) *	-0.2 (-0.2,-0.1) *	-0.3 (-0.4,-0.3) *	-0.2 (-0.2,-0.1) *	-0.2 (-0.3,-0.2) *	-0.1 (-0.2,-0.1) *
	**50-59**	-0.3 (-0.4,-0.3) *	-0.2 (-0.2,0.1) *	-0.3 (-0.3,-0.2) *	-0.2 (-0.2,-0.1) *	-0.3 (-0.3,-0.2) *	-0.1 (-0.2,-0.1) *
	**60-69**	-0.3 (-0.3,-0.2) *	-0.2 (-0.2,-0.1) *	-0.2 (-0.3,-0.2) *	-0.2 (-0.2,-0.1) *	-0.2 (-0.3,-0.1) *	-0.1 (-0.2,-0.1) *
	**70-79**	-0.3 (-0.3,-0.2) *	-0.2 (-0.2,-0.1) *	-0.2 (-0.3,-0.2) *	-0.2 (-0.2,-0.1) *	-0.1 (-0.2,-0.1) *	-0.1 (-0.1,0.1)
	**≥80**	-0.2 (-0.3,-0.2) *	-0.2 (-0.2,-0.1) *	-0.2 (-0.3,-0.1) *	-0.2 (-0.3,-0.1) *	-0.2 (-0.3,0.1)	-0.1 (-0.3,0.1)
**non-HDL cholesterol** (mmol/l)	**20-39**	0.1 (0.0,0.1)	0.3 (0.2,0.4) *	0.1 (-0.1,0.2)	0.3 (0.1,0.4) *	-0.1 (-0.2,0.1)	0.1 (-0.1,0.4)
	**40-49**	0.2 (0.1,0.2) *	0.3 (0.2,0.3) *	0.1 (0.1,0.2) *	0.2 (0.1,0.2) *	0.1 (-0.1,0.2)	0.1 (0.0,0.2)
	**50-59**	0.1 (0.1,0.2) *	0.1 (0.1,0.2) *	0.1 (-0.1,0.2)	-0.1 (-0.1,0.0)	0.1 (-0.1,0.3)	-0.1 (-0.2,0.1)
	**60-69**	-0.1 (-0.1,0.1)	-0.1 (-0.2,-0.1) *	0.1 (-0.1,0.1)	-0.1 (-0.2,0.1)	-0.1 (-0.2,0.1)	-0.1 (-0.3,0.1)
	**70-79**	-0.1 (-0.2,-0.1) *	-0.1 (-0.2,-0.1) *	0.1 (-0.1,0.2)	-0.1 (-0.2,0.1)	-0.1 (-0.2,0.1)	-0.1 (-0.3,0.1)
	**≥80**	-0.1 (-0.2,-0.1) *	-0.1 (-0.2,-0.1) *	-0.1 (-0.3,0.2)	0.1 (-0.3,0.4)	-0.4 (-0.8,0.1)	-0.1 (-0.5,0.4)
**Triacylglycerol** (mmol/l)	**20-39**	1.1 (1.0,1.3) *	2.0 (1.9,2.1) *	0.9 (0.4,1.3) *	1.5 (1.1,1.9) *	0.4 (0.2,0.6) *	0.8 (0.5,1.2) *
	**40-49**	1.1 (1.0,1.1) *	1.6 (1.5,1.7) *	0.7 (0.6,0.8) *	0.9 (0.8,1.1) *	0.4 (0.2,0.6) *	0.5 (0.4,0.6) *
	**50-59**	0.9 (0.9,1.0) *	1.1 (1.0,1.1) *	0.6 (0.5,0.7) *	0.7 (0.5,0.8) *	0.4 (0.2,0.6) *	0.3 (0.1,0.5) *
	**60-69**	0.7 (0.6,0.7) *	0.7 (0.6,0.7) *	0.6 (0.3,0.9) *	0.3 (0.1,0.5) *	0.2 (-0.1,0.3)	0.3 (0.1,0.6) *
	**70-79**	0.5 (0.5,0.6) *	0.5 (0.4,0.5) *	0.6 (0.4,0.8) *	0.4 (0.2,0.6) *	0.1 (-0.1,0.2)	0.1 (-0.1,0.3)
	**≥80**	0.4 (0.3,0.4) *	0.4 (0.3,0.4) *	0.4 (0.2,0.6) *	0.5 (0.3,0.7) *	0.1 (-0.5,0.8)	0.2 (-0.1,0.4)

* indicates $p<0.05$

Data are mean (95% CI) differences in risk factor levels between people T2DM and controls without diabetes, stratified by ethnic group and sex (e.g. level in White women with T2DM minus level in White women controls). Differences are adjusted for between group differences in age, sex, general practice and deprivation.

ESM Fig 1. Combining ethnicity records from CPRD and hospital records (HES)

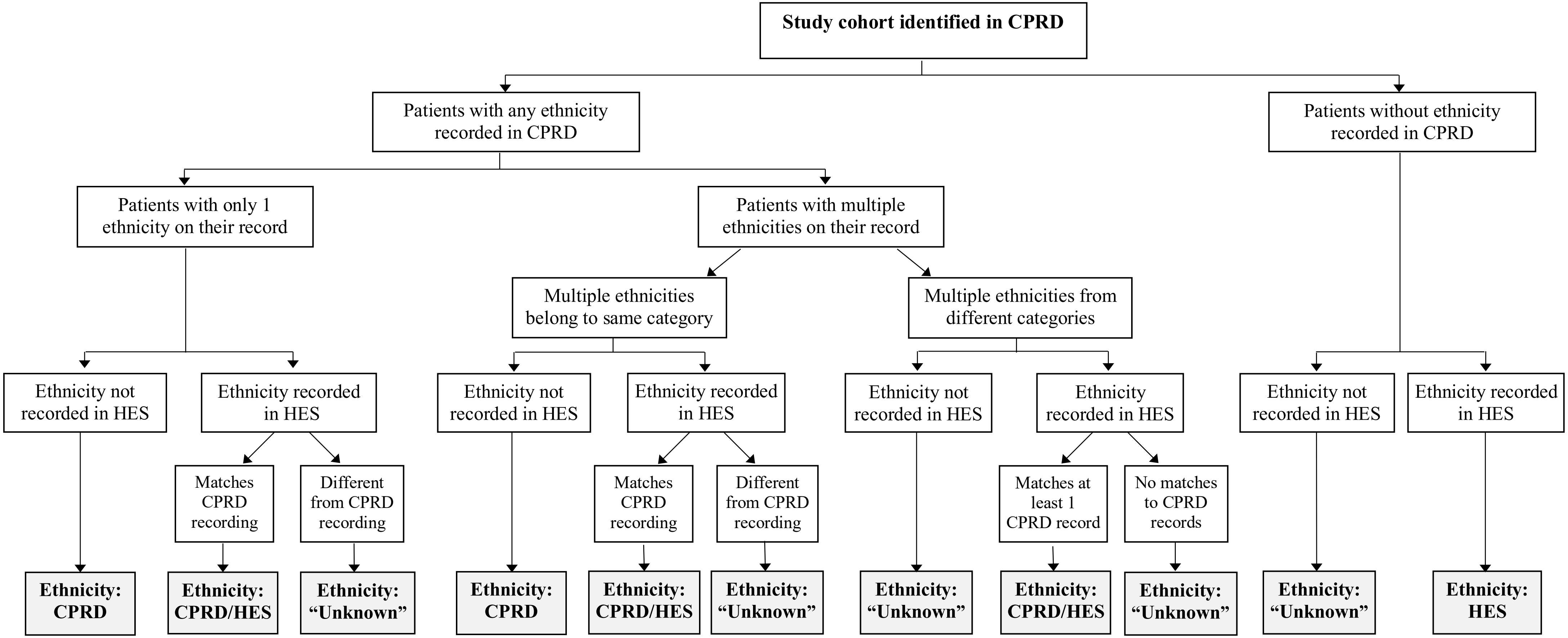

Supplement: Supplementary file 1 — (PDF 870 kb) [file 125_2020_5169_MOESM1_ESM.pdf]
